# Mitochondrial ROS control neuronal excitability and cell fate in frontotemporal dementia

**DOI:** 10.1002/alz.12394

**Published:** 2021-05-31

**Authors:** Noemí Esteras, Olga Kopach, Marta Maiolino, Vincenzo Lariccia, Salvatore Amoroso, Seema Qamar, Selina Wray, Dmitri A. Rusakov, Morana Jaganjac, Andrey Y. Abramov

**Affiliations:** ^1^ Department of Clinical and Movement Neurosciences UCL Queen Square Institute of Neurology London UK; ^2^ Department of Clinical and Experimental Epilepsy UCL Queen Square Institute of Neurology London UK; ^3^ Department of Biomedical Sciences and Public Health School of Medicine University “Politecnica delle Marche,” Ancona Italy; ^4^ Department of Clinical Neurosciences Cambridge Institute for Medical Research University of Cambridge Cambridge UK; ^5^ Department of Neurodegenerative Disease UCL Queen Square Institute of Neurology London UK; ^6^ Qatar Analytics & BioResearch Lab Anti‐Doping Lab Qatar Doha Qatar; ^7^ Division of Molecular Medicine Rudjer Boskovic Institute Zagreb Croatia

**Keywords:** 4R tau, AMPA receptors, calcium signaling, frontotemporal dementia, glutamate, induced pluripotent stem cells, MAPT 10+16, mitochondrial antioxidants, mitochondrial reactive oxygen species, NMDA receptors, tau

## Abstract

**Introduction:**

The second most common form of early‐onset dementia—frontotemporal dementia (FTD)—is often characterized by the aggregation of the microtubule‐associated protein tau. Here we studied the mechanism of tau‐induced neuronal dysfunction in neurons with the FTD‐related 10+16 *MAPT* mutation.

**Methods:**

Live imaging, electrophysiology, and redox proteomics were used in 10+16 induced pluripotent stem cell‐derived neurons and a model of tau spreading in primary cultures.

**Results:**

Overproduction of mitochondrial reactive oxygen species (ROS) in 10+16 neurons alters the trafficking of specific glutamate receptor subunits via redox regulation. Increased surface expression of α‐amino‐3‐hydroxy‐5‐methyl‐4‐isoxazolepropionic acid (AMPA) and N‐methyl‐D‐aspartate (NMDA) receptors containing GluA1 and NR2B subunits leads to impaired glutamatergic signaling, calcium overload, and excitotoxicity. Mitochondrial antioxidants restore the altered response and prevent neuronal death. Importantly, extracellular 4R tau induces the same pathological response in healthy neurons, thus proposing a mechanism for disease propagation.

**Discussion:**

These results demonstrate mitochondrial ROS modulate glutamatergic signaling in FTD, and suggest a new therapeutic strategy.

## Graphical abstract

**Tau‐induced mitochondrial** reactive oxygen species (**ROS) promote** α‐amino‐3‐hydroxy‐5‐methyl‐4‐isoxazolepropionic acid receptor (**AMPAR)/**N‐methyl‐D‐aspartate receptor (**NMDAR)‐mediated Ca^2+^‐excitotoxicity and neuronal death in 4R tauopathies**. 10+16 *MAPT* mutation induces an increase in 4R tau and is causative of frontotemporal dementia. 10+16 *MAPT* induced pluripotent stem cell (iPSC)‐derived neurons either from patients or genetically engineered, present mitochondrial hyperpolarization resulting in increased mitochondrial ROS production compared to healthy donors or their isogenic wild‐type tau counterpart. Our results indicate that mitochondrial ROS overproduction promotes an increase in the surface levels of specific subunits (NR2B and GluA1) of NMDAR and AMPAR, probably via the oxidation of specific peptides and proteins involved in their trafficking. This leads to the upregulation of glutamate‐induced calcium signaling, resulting in calcium overload and ultimately cell death. Treatment of neurons with the mitochondrial antioxidants MitoQ or MitoTEMPO reduces the surface levels of these NMDAR and AMPAR subunits, avoids the glutamate‐induced calcium overload, and protects against excitotoxicity. Importantly, extracellular 4R tau, either secreted by the iPSC neurons from patients or the recombinant K18 fragment, exert the same calcium deregulation in control neurons, which can also be prevented with mitochondrial antioxidants. These data suggest an additional role for extracellular tau in the spread of the disease and the neuronal dysfunction.

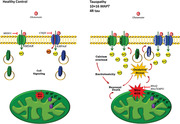

## 1 BACKGROUND

Mutations in the *MAPT* gene, encoding the microtubule‐associated protein tau, are known to cause familial frontotemporal dementia (FTD), the second most common cause of early onset dementia.^1^ Among them, the intronic 10+16 mutation causes augmented splicing in of *MAPT* exon 10 and therefore an increase in the proportion of 4R‐tau isoforms (containing four repeats of the microtubule‐binding domain) versus 3R isoforms (containing three repeats), which are otherwise balanced in the adult brain. Changes in the tau isoforms ratio are sufficient to cause neurodegeneration in this and other disorders, by a mechanism that is not fully understood. Altered regulation of microtubule dynamics,^2^ neuroinflammatory mediators,^3^ calcium deregulation,^4^, ^5^, ^6^ as well as oxidative stress and mitochondrial dysfunction,^7^, ^8^ have been all suggested as potential mechanisms of tau‐related neuronal death.

Although neurodegeneration is a hallmark of dementia, memory decline appears prior to neuronal loss in a number of neurodegenerative disorders, including FTD. Synaptic plasticity is thought to represent a key mechanism underpinning learning and memory. Indeed, synaptic dysfunction is the best correlate with the progression of cognitive decline in tauopathies such as Alzheimer's disease (AD).^9^

**RESEARCH IN CONTEXT**

1. **Systematic review**: Tau is involved in a number of neurodegenerative disorders such as frontotemporal dementia. Different mechanisms have been described to understand its role in neurodegeneration, including synaptic dysfunction, mitochondrial alterations, oxidative stress, and calcium deregulation.
2. **Interpretation**: Our findings demonstrate that tau impairs glutamatergic signaling via mitochondrial reactive oxygen species (ROS) overproduction, leading to the overactivation of N‐methyl‐D‐aspartate (NMDA) and α‐amino‐3‐hydroxy‐5‐methyl‐4‐isoxazolepropionic acid (AMPA) glutamate receptors, which results in excessive calcium entry to the cytosol and neuronal death. We demonstrate the underlying mechanism involves the trafficking of specific glutamate receptor subunits and the possibility to revert these effects using mitochondrial antioxidants.
3. **Future directions**: We propose that mitochondria, through mitochondrial ROS, modulate glutamatergic signaling in neurons, with tau‐triggered overproduction of mitochondrial ROS leading to neuropathological effects. Future experiments will extrapolate these findings to other tauopathies and neurodegenerative disorders in which different protein aggregates are the hallmark (amyloid beta, tdp‐43, alpha‐synuclein); and investigate the protective effect of mitochondrial antioxidants as a therapeutic target.

Glutamate is the main excitatory neurotransmitter in the central nervous system acting through either ionotropic glutamate receptors (plasmalemmal ion channels such an N‐methyl‐D‐aspartate [NMDA] and α‐amino‐3‐hydroxy‐5‐methyl‐4‐isoxazolepropionic acid [AMPA] receptors that mediate Ca^2+^ and Na^+^ influx) or metabotropic glutamate receptors (mGlu, protein G‐coupled receptors that mediate Ca^2+^ release from intracellular stores, among other signaling pathways). However, excessive activation of glutamate receptors prompts hyperexcitability in neurons, resulting in intracellular calcium overload that triggers a pathological process known as excitotoxicity, which ultimately leads to neuronal death.^10^, ^11^, ^12^ Impaired glutamatergic transmission has been previously demostrated in tauopathies, pointing to tau‐induced dysfunction of NMDA and AMPA receptors.^13^, ^14^

Reactive oxygen species (ROS) are able to modulate physiological and pathological signal transduction in the brain. NMDA glutamate receptor activation can trigger superoxide production by nicotinamide adenine dinucleotide phosphate (NADPH) oxidase, contributing to cell signaling, but also to neuronal damage.^15^, ^16^ Conversely, the function of NMDA receptors can also be modified by ROS, impairing synaptic function in AD.^17^, ^18^ Specifically, ROS produced by the mitochondria regulate diverse physiological processes in brain cells, including signal transduction,^19^, ^20^, ^21^ but overproduction of mitochondrial ROS can trigger cellular dysfunction and neuronal death.^22^

**HIGHLIGHTS**

- Tau impairs glutamatergic signaling in frontotemporal dementia (FTD) via mitochondrial reactive oxygen species (ROS) overproduction.
- Tau‐induced mitochondrial ROS alter the trafficking of specific glutamate receptors.
- Mitochondrial antioxidants prevent calcium overload and excitotoxicity in FTD.
- Extracellular 4R tau impairs glutamatergic signaling by the same mechanism.

Indeed, our previous studies in induced pluripotent stem cell (iPSC)–derived neurons with the FTD‐related 10+16 *MAPT* mutation show that mitochondrial ROS overproduction is a key pathological event of tau‐induced pathology.^8^ Mitochondrial dysfunction in these cells leads to oxidative stress and neuronal death that can be prevented with mitochondrial antioxidants. In addition, 10+16 neurons are more vulnerable to calcium overload^5^, ^23^ and exhibit severe functional impairments including a depolarized resting membrane potential and changed neuronal excitability due to reduced Na_v_1.6 expression.^24^ Here, we have tried to understand how this system of impairments interact in the course of neurodegeneration, by exploring a possible link between them and the dysregulation of the glutamatergic signaling. We have found that overproduction of ROS in the mitochondria of the 10+16 neurons leads to an increase in the surface levels of specific subunits of NMDA and AMPA receptors via protein oxidation. This impairs the glutamate‐induced signal transduction leading to calcium overload. Supplementation of the cells with mitochondrial antioxidants completely recovers the glutamate‐induced calcium response in patients’ neurons and protects against excitotoxicity. Similar results were obtained in isogenic‐engineered 10+16 *MAPT* iPSC‐derived neurons and in primary neurons treated with extracellular 4R tau. Our results highlight a direct link among mitochondrial dysfunction, oxidative stress, and calcium deregulation in the mechanism of 4R tau‐induced neuronal death, which is not restricted to FTD but can be extrapolated to other forms of dementia. These findings demonstrate a key role for mitochondria in pathophysiological signaling and the possibility to modulate its effects with mitochondrial antioxidants.

## 2 METHODS

### 2.1 Materials

Unless otherwise specified, all the materials were obtained from Thermo Fisher Scientific (Life Technologies). NMDA, AMPA, MitoTEMPO, Trolox, and MK801 were obtained from Sigma‐Aldrich. Kainic acid, CNQX, NBQX, SYM2206 were obtained from Tocris. Tau 2N3R and 2N4R are from Abcam. Deuterated polyunsaturated fatty acid (D‐PUFA) 11,11,14,14‐D_4_‐α‐linoleic acid, was kindly provided by Dr. Mikhail S. Shchepinov (Retrotope, Inc), and its synthesis was previously described.^25^ MitoQ was kindly provided by Dr. Michael P. Murphy (Medical Research Council Mitochondrial Biology Unit).

### 2.2 Human iPSC‐derived cortical neuron cultures

Controls 1‐3 and patients 1‐2 iPSC lines were generated by retroviral‐transduction reprogramming of fibroblasts. All the information regarding the reprogramming of the fibroblasts and characterization of these lines was published previously.^26^ Control 1 was obtained from the laboratory of Dr. Tilo Kunath, control 2 from the Coriell repository, and control 3 was purchased from Thermo Fisher Scientific. The two iPSC lines with the 10+16 *MAPT* mutation were generated from fibroblasts obtained from the National Hospital for Neurology and Neurosurgery, London, UK. In addition, we used a genetically engineered *MAPT* IVS10+16^–/+^ line which was generated using zinc finger nuclease (ZFN) technology to introduce the *MAPT* monoallelic 10+16 mutation into a parental iPSC line (Sigma) as described in Verheyen et al.^27^ The control line in these experiments is wild type (wt) at tau locus, has a green fluorescent protein reporter inserted into *SLC17a7* gene using ZFN technology, and was generated from the same parental line as IVS10+16^–/+^, thus both have the same genetic background. Both lines are available via the EBISC repository (SIGi001‐A‐1 and SIGi001‐A‐13).

Differentiation of the iPSC lines into cortical neurons was done using the protocol described by Shi et al.^28^ Briefly, cells were subjected to 10 days of dual SMAD inhibition with 1 μM dorsomorphin (Tocris) and 10 μM SB431542 (Tocris), followed by extended neurogenesis in N2B27 media. Around 40 days after neural induction, cells were plated in poly‐ornithine/laminin (Sigma) coated μ‐Slide 8 well Ibidi chambers (Thistle Scientific) for most of the experiments, or glass coverslips similarly coated for carrying out electrophysiological experiments. Cells were maintained in neural maintenance media^28^ with media changes twice a week. All the experiments were performed in neurons older than 120 days (after induction).

### 2.3 Primary neuronal‐astrocytic co‐cultures

Primary cortical co‐cultures of neurons and astrocytes were prepared as described previously, from the cortex of Sprague‐Dawley rat pups (P2‐P4) from the University College London breeding colony. Experimental procedures were performed in full compliance with the United Kingdom Animal (Scientific Procedures) Act of 1986 and with the European directive 2010/63/EU. Cortex was rapidly removed into ice‐cold phosphate‐buffered saline, and the tissue was trypsinized (0.05% trypsin/ethylenediaminetetraacetic acid) for 15 minutes at 37°C, homogenized and plated on poly‐D‐lysine‐coated glass coverslips. Cultures were maintained at 37°C in a humidified atmosphere of 5% CO_2_ in Neurobasal A media supplemented with B27 and 2 mM Glutamax and in the presence of penicillin/streptomycin. Media was replaced after one week and cells were used at 12‐15 DIV in all the experiments.

### 2.4 K18 Tau protein expression and purification

Recombinant K18 Tau protein was expressed in BL21(DE3) *E.coli* cell line transformed with pJ414 vector containing a bacterial codon optimized synthetic gene encoding I260C/C291A/C322A tau K18. Protein was purified as described previously^29^ by incubating the cell lysates containing K18 protein at 90°C to precipitate majority of the *E.coli* proteins leaving the Tau in solution. Protein was further purified on a SP Sepharose column followed by a Superdex 75 size exclusion chromatography step using an Akta Chromatography System (GE Healthcare).

### 2.5 Live cell imaging

Most of the experiments were performed in Hanks’ balanced salt solution (HBSS) buffer (with Ca^2+^ and Mg^2+^) supplemented with 10 mM HEPES and adjusted to pH 7.4. In specific experiments, HBSS Ca^2+^‐free media (consisting of commercial HBSS Ca^2+^‐Mg^2+^ free, supplemented with 2 mM MgCl_2_ and 0.5 mM ethylene glycol tetraacetic acid) or HBSS Mg^2+^‐free media (consisting of commercial HBSS Ca^2+^‐Mg^2+^ free, supplemented with 2 mM CaCl_2_) were used.

#### 2.5.1 Ratiometric cytosolic Ca^2+^ imaging

[Ca^2+^]_c_ was monitored in single cells using Fura‐2 AM, a high affinity intracellular calcium indicator which is ratiometric and allows an accurate measurement of the cytosolic Ca^2+^ as the ratio of the emissions of the dye in response to 340/380 excitation, independently of loading variations. Cells were loaded for 30 minutes at room temperature with 5 μM fura‐2 AM in the presence of 0.005% pluronic in HBSS buffer. Fluorescence measurements were made on an epifluorescence inverted microscope equipped with a 20x fluorite objective (Nikon Eclipse Ti‐S). Excitation light was provided by a xenon arc lamp, the beam passing a monochromator at 340 and 380 nm (Cairn Research). Emitted fluorescence light was reflected through an ET510/80 m filter to an Andor Zyla sCMOS camera (Cairn Research) and digitized to a 16‐bit resolution. All imaging data were collected and analyzed using Andor iQ2 (Andor) and Origin Pro 2018 (Origin Lab) software. Area under the curve (AUC; mathematical area) was calculated with the integration function in Origin Pro 2018, using a constant number of frames and after baseline subtraction.

#### 2.5.2 Two‐photon (2P) excitation fluorescent imaging

Cells were bolus loaded with the cell‐permeable Ca^2+^ indicator Oregon Green BAPTA‐1 (OGB‐1 AM; 5 μM, Invitrogen) by incubation for 30 minutes at 37°C. After loading, the cells were washed for approximately 30 minutes for de‐etherification of the dye. A sample was placed in a recording chamber mounted on the stage of an Olympus BX51WI upright microscope equipped with galvo scanners, and integrated with patch‐clamp electrophysiology. 2P excitation microscopy was carried out using an Olympus FV1000 imaging system optically linked to a Ti:Sapphire MaiTai femtosecond‐pulse laser (SpectraPhysics‐Newport) at λ2Pex = 800 nm (optimized for OGB‐1) with appropriate emission filters. For the time‐lapse changes in OGB‐1 fluorescence, images were collected using 512 × 512 pixel frames in the stream acquisition mode. Various digital zooms were used to collect images at high resolution. Recordings were carried out in a bicarbonate‐buffered Ringer solution containing (in mM) 126 NaCl, 3 KCl, 2 MgSO_4_, 2 CaCl_2_, 26 NaHCO_3_, 1.25 NaH_2_PO_4_, 10 D‐glucose, saturated with 95% O_2_ and 5% CO_2_ (pH 7.4; 300‐310 mOsmol). To avoid phototoxic damage to the cells during scanning, the laser power was always kept at its minimum. To provide a brief, localized agonist application, glutamate (5 μM) was applied to the cells via a fabricated glass micropipette (≈1 μm the tip diameter). To enable visualization of the area and duration of the agonist spread, the fluorescent tracer Alexa Fluor‐594 (100 μM) was added into the pipette.^30^ Changes in [Ca^2+^]_c_ were expressed as the changes in OGB‐1 fluorescence at the maximum of the fluorescent signal over the baseline (ΔF/F_0_) as earlier.

#### 2.5.3 Mitochondrial ROS production

Mitochondrial ROS production was analyzed using the mitochondrial‐targeted dye MitoTracker Red CM‐H_2_XRos. Cells were loaded with 1 μM dye for 20 minutes at room temperature and images were taken on the confocal microscope Zeiss 710 LSM with an integrated META‐detection system using a 40x oil‐immersion objective. The dye was excited at 561 nm, and the emitted fluorescence was detected above 580 nm. Imaging of the cells was recorded for several minutes and the rate of increase in red fluorescence was then analyzed using Zen (Zeiss) and Origin Pro 2018 (Origin Lab) software.

#### 2.5.4 Mitochondrial membrane potential

Mitochondrial membrane potential (ΔΨm) was analyzed as previously described.^31^ Cells were loaded for 40 minutes with 25 nM tetramethylrhodamine methyl ester (TMRM, Sigma). Z‐stacks were acquired using a Zeiss 710 VIS CLMS confocal microscope equipped with a META detection system and an x40 oil immersion objective (Zeiss). The dye was excited at 561 nm, and the emitted fluorescence was detected above 580 nm. Z‐stacks were analyzed and average intensity was calculated using Volocity 3D Image Analysis Software (PerkinElmer).

#### 2.5.5 Cell death

Cells were loaded for 20 minutes at room temperature with 20 μM propidium iodide (PI; only permeable to dead cells) and 10 μM Hoechst 33342 (which stains chromatin in all cells). Images of the cells were then acquired using an epifluorescence inverted microscope equipped with a 20x fluorite objective (Nikon Eclipse Ti‐S). Excitation light was provided by a xenon arc lamp, the beam passing a monochromator at 340 or 530 nm. Emitted fluorescence light was reflected through an ET455/50 m or a ET605/52 m filter, respectively, to an Andor Zyla sCMOS camera. Images were analyzed using ImageJ software by counting PI‐positive cells (dead) relative to the total number of cells (stained by Hoechst). A total number of 300 to 1000 cells were counted per field in n = 10 to 19 different fields. Experiments were repeated 3 to 4 times with different cell preparations.

#### 2.5.6 Apoptosis

Caspase‐3 activation was assessed by live confocal imaging after loading the cells for 15 minutes with 10 μM NucView 488 caspase‐3 subtrate (Biotium) and 10 μM Hoechst 33342. Bright NucView positive nuclei colocalizing with Hoechst were considered apoptotic cells.

### 2.6 Cross‐linking assay

To estimate the trafficking of the different AMPA receptor (AMPAR) and NMDA receptor (NMDAR) subunits between the cytosol and the cellular membrane we performed a crosslinking assay with the membrane impermeable crosslinker bis(sulfosuccinimidyl)suberate BS3 (ThermoFisher) as described by Boudreau et al.^32^ Briefly, BS3 forms a covalent bond between the cell surface proteins in close proximity leaving intracellular proteins unaffected. Therefore, the apparent molecular weight of the receptors located in the cell membrane increases compared to the non‐crosslinked (intracellular) ones, and it is possible to distinguish and quantify each fraction using western blot. A sample not treated with BS3 was included to confirm the position of the intracellular (non cross‐linked) band. The absence of higher molecular weight bands in the actin blot serves as a technical control indicating that intracellular proteins were not crosslinked.

When indicated, 100 nM MitoTEMPO was added to the cell media 2 hours before the experiment. Neurons were then washed, the crosslinking reaction was performed incubating them in 0.5 mM BS3 in HBSS for 15 minutes at 37°C and terminated by adding 100 mM glycine (10 minutes at 4°C). Solution was washed and protein extracts collected in ice‐cold RIPA lysis buffer supplemented with protease and phosphatase inhibitors (Thermo Fisher). Samples were snap frozen, sonicated, and centrifuged at 14000 rpm; and protein content of the extracts was determined by the Pierce BCA protein assay (Thermo Fisher).

### 2.7 Western blot

Fifteen to twenty μg of protein extracts were then fractionated on a sodium dodecyl sulfate polyacrylamide gel (4%–12%; Thermo Fisher), transferred to a polyvinylidene difluoride membrane (Bio‐Rad) and blocked with 5% non‐fat milk. Membranes were incubated overnight with the corresponding primary antibodies diluted in 5% bovine albumin serum: pan AMPA (mabn832) 1:1000 from Merck Millipore; NR1 (SAB4501301) 1:1000 from Sigma‐Aldrich; GluA1 (ab109450) 1:2000 and NR2B (ab28373) 1:500 from Abcam; beta‐actin (4970) 1:5000 from Cell Signaling Technologies; beta‐III Tubulin (MAB1195) 1:5000 from R&D Systems; and afterward with the corresponding specie‐specific horseradish peroxidase (HRP)‐conjugated secondary antibodies. The luminol‐based Pierce ECL Western Blotting Substrate (Thermo Fisher Scientific) was used to detect the HRP activity. Protein band densities were quantified using Image J (NIH) after scanning the X‐ray films. For the estimation of total levels of the protein of interest in the crosslink experiments, intracellular and surface band densities were added. In some cases, western blots with non‐crosslinked proteins were performed. To compare the results between experiments, in all cases results were normalized to control.

### 2.8 Redox proteomics

For redox proteomics, protein extracts were prepared from cell cultures on RIPA buffer and separated on Bolt 4%‐12% Bis‐Tris Plus polyacrylamide gels to minimize artefactual protein modifications. The whole sample lanes excised were divided into eight equal parts. Samples were reduced with 10 mM dithiothreitol, alkylated with 10 mM iodoacetamide and digested with the mass spectrometry grade mixture of trypsin and Lys‐C (trypsin/Lys‐C, Promega) according to the manufacturer's protocol. To minimize the interference of artifactual protein oxidation all samples were processed in parallel. Complex peptide mixtures were loaded on a 25 cm reversed‐phase C18 column (75 μm, 2 μm Acclaim RSLC C18, Thermo Scientific) using nano Easy n‐LC II (Thermo Scientific) system coupled to an Orbitrap Elite mass spectrometer (Thermo Scientific). Peptides were separated over a 90 minute linear gradient from 5% acetonitrile, 0.1% formic acid to 37% acetonitrile, 0.1% formic acid with a constant flow of 300 nL/min, followed by a wash step and re‐equilibration prior to injection of new sample. The Orbitrap Elite operated in a data‐dependent mode using collision‐induced dissociation (CID) for peptide fragmentation as described previously.^33^

The raw MS data files were searched against Uniprot Homo sapiens database (downloaded on 12th October 2017) using Sequest HT search engine in Proteome Discoverer 2.2 (Thermo Fisher Scientific), with false discovery rate (FDR) calculated by a target–decoy approach set to 0.01. Two Sequest HT searches were performed where trypsin or Lys‐C, respectively, were selected as the enzyme. The following search parameters were used: 10 ppm precursor mass tolerances, 0.6 Da fragment mass tolerance, and maximum of two missed cleavages with a minimum peptide length of six amino acids. Other parameters include carbamidomethylation of cysteine (+57.021 Da) as a static modification and several variable modifications of peptides: acetylation of Lys (+42.011 Da), amino acid mono‐oxidation (+15.995 Da), di‐oxidation (+31.990 Da), tri‐oxidation (+47.985 Da), deamidation (+0.984 Da), nitration of Trp or Tyr (+44.985 Da), oxidation of His to Asn (–23.016 Da) or Asp (–22.032 Da), oxidation of Lys to aminoadipic acid (+14.963 Da), Trp oxidation to oxolactone (+13.979 Da), conversion of Trp to kynurenine (+3.995 Da), carbonylation of Arg to glutamic semialdehyde (GluSA, –43.053 Da), Lys to aminoadipic semialdehyde (Allysine, –1.032 Da), and Pro to pyrrolidinone (–30.010 Da).

The mass spectrometry proteomics data have been deposited to the ProteomeXchange Consortium via the PRIDE^34^ partner repository with the dataset identifier PXD025083.

### 2.9 Electrophysiology

A sample of human iPSC‐derived cortical neurons was placed in a recording chamber mounted on the stage of an Olympus BX51WI upright microscope equipped with a LUMPlanFI/IR 40×0.8 objective coupled to an infrared DIC imaging system. Electrophysiological recordings were performed using Multipatch 700B amplifier controlled by pClamp 10.2 software package (Molecular Devices). Recordings were made in a bicarbonate‐buffered solution (aCSF) containing (in mM) 126 NaCl, 3 KCl, 2 MgSO_4_, 2 CaCl_2_, 26 NaHCO_3_, 1.25 NaH_2_PO_4_, 10 D‐glucose (95% O_2_ and 5% CO_2_; pH 7.4; osmolarity 300‐310 mOsmol) at 31°C to 33°C. Recording electrodes (the resistance of 3.5–5 MΩ) were filled with an intracellular solution containing (in mM) 126 K‐gluconate, 10 HEPES, 4 KCl, 4 MgCl_2_, 2 BAPTA, 4 Mg‐ATP, 0.4 GTP‐Na (pH 7.2 with KOH, osmolarity ≈290 mOsmol). Neurons were monitored for spontaneous firing activity in either cell‐attached configuration (after formation of gigaseal) or whole‐cell (at –60 mV). Once after membrane breakthrough (whole‐cell), cells were monitored for the intrinsic passive membrane properties, including the resting membrane potential (V_rest_), capacitance (C_m_), and input resistance (R_in_), as described by Kopach et al.^24^ Synaptic activity (excitatory transmission) was examined by recording spontaneous excitatory postsynaptic currents (sEPSCs) at –70 mV. The glutamate‐evoked currents were recorded at different membrane potential by applying exogenous glutamate using the rapid‐application system. An application pipette was positioned close to a recorded neuron, and glutamate (100 μM in aCSF) was applied in a series of 500‐ms pulses (or as indicated), in varied inter‐pulse intervals, with a pressure supplied by a two‐channel PDES‐02DX pneumatic micro ejector system (npi electronic GmbH). Glutamate‐evoked currents were analyzed for the peak amplitude calculated as mean amplitude for train of repetitively evoked responses (at least 3 to 5 trials), in every tested cell. Antagonists (10 μM CNQX, 10 μM NBQX, 10 μM MK801) were applied in bath.

Clampfit 10.3 software (Molecular Devices) and OriginPro (Origin Lab) were used for analysis. Mini Analysis Program (Synaptosoft) was used for off‐line analysis of sEPSCs, as described.^35^ Briefly, excitatory events were distinguished from baseline noise by setting the appropriate parameters for each individual cell and by manually eliminating false events. The AMPAR‐mediated postsynaptic currents were analyzed for the frequency of their occurrence and the amplitude. For bursting activity, polysynaptic events occurring in the bursts were counted.

### 2.10 Statistical analysis

OriginPro 2019 and IBM Statistics SPSS 25 were used for the statistical analysis. Data sets were first probed for normality with the Shapiro‐Wilk test and homogeneity of variances was analyzed with Levene's test. In most of the cases, the data sets did not show a normal distribution; therefore, non‐parametric tests were performed (Kruskal‐Wallis H test or Mann‐Whitney U‐test). When appropriate, Student's t‐test or analysis of variance followed by Bonferroni post‐hoc test were performed to estimate the statistical significance between experimental groups. Histograms represent the mean ± standard deviation, and box plots represent the median and 25 and 75 percentiles, with the distribution profiles showing single‐cell values, unless otherwise indicated. Electrophysiological data are presented as mean ± standard error of the mean with n referring to the number of cells analyzed. The Fisher exact test was used to determine statistical difference between the two category variables as indicated in the text. A *P*‐value less than 0.05 was considered statistically significant for either test.

## 3 RESULTS

### 3.1 Dysfunction of human iPSC‐derived neurons with the MAPT 10+16 mutation links to upregulation of AMPAR and NMDAR

We have previously shown that human iPSC‐derived cortical neurons with the 10+16 *MAPT* mutation linked to FTD exhibit pathophysiological excitability at late stages of neurogenesis (≈150 DIV), revealed as a depolarized resting membrane potential associated with impaired firing due to reduced expression of Nav1.6 channels.^24^ We therefore used electrophysiology to further explore dysfunctions on these neurons.

**FIGURE 1:**
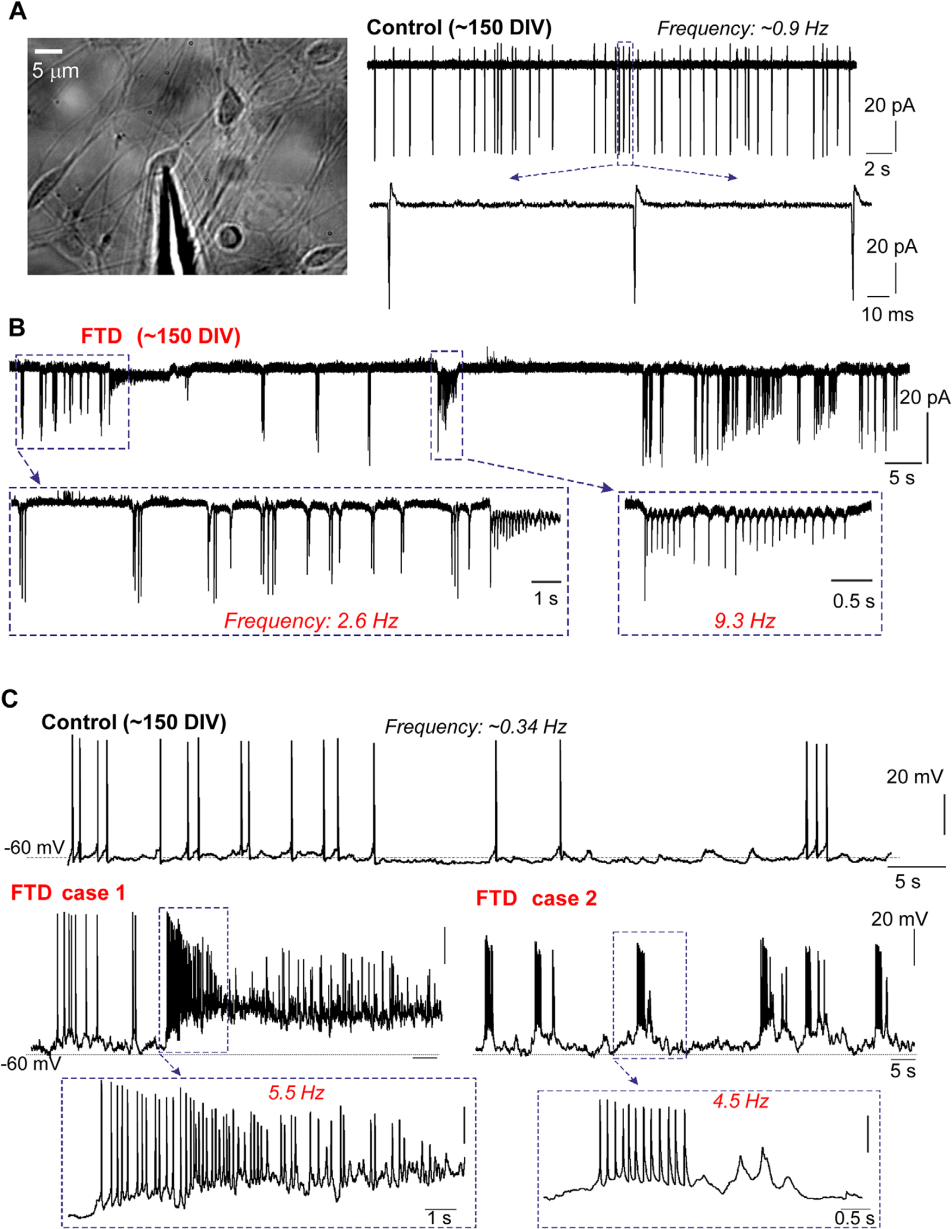
Human induced pluripotent stem cell (iPSC)‐derived cortical neurons display pathological activity in frontotemporal dementia (FTD)‐related *MAPT* 10+16 mutation. A, Image, human iPSC‐derived cortical neurons at ≈150 DIV for electrophysiology. Traces, typical firing activity recorded under cell‐attached mode from iPSC‐derived cortical neurons in control lines, showing spontaneous action potentials (AP), evoked at the frequency ≈0.9 Hz. Lower row shows individual AP spikes on an expanded scale (*N* = at least 8 cells from 4 independent preparations). B, Representative spontaneous AP firing pattern in iPSC‐derived neurons from FTDP group, showing irregular firing activity and bursts of spontaneous AP. Boxes depict an area for illustration on an expanded scale, with the corresponding frequency of discharge shown (*N* = 8 cells, 2 independent preparations). C, Examples of spontaneous AP firing (whole‐cell, Vm = –60 mV) in iPSC‐derived cortical neurons (≈150 DIV) from control lines (upper row) and FTDP with the *MAPT* 10+16 mutation (lower traces). Two individual cases illustrated; boxes depict an area on an expanded scale, with the corresponding frequency of discharge

In cell‐attached mode, iPSC‐derived cortical neurons (healthy cohort) demonstrated a sustained spontaneous activity, a constant and regular firing (Figure 1A), representing genuine human cell activity (i.e., not compromised by membrane breakthrough and potential washing out/dilution of intracellular regulators). On the contrary, iPSC‐derived neurons with the 10+16 *MAPT* mutation displayed bursting, irregular discharge consisting of high‐frequency bursts (≈2.6 Hz to 9.3 Hz), followed by periods of prolonged “silence” (Figure 1B) associated with membrane depolarization (Figure S1A in supporting information). In whole‐cell mode, iPSC‐derived neurons with the 10+16 *MAPT* mutation (held at –60 mV) revealed bursts of spontaneous discharge, again, opposing regular firing in the age‐matched control neurons (Figure 1C). We observed two characteristic patterns of spontaneous activity in FTD: (1) high‐frequency firing associated with sustained depolarization (case 1) and (2) trains of short bursts consisted of few spikes followed by periods of “silence” (case 2; Figure 1C).

**FIGURE 2:**
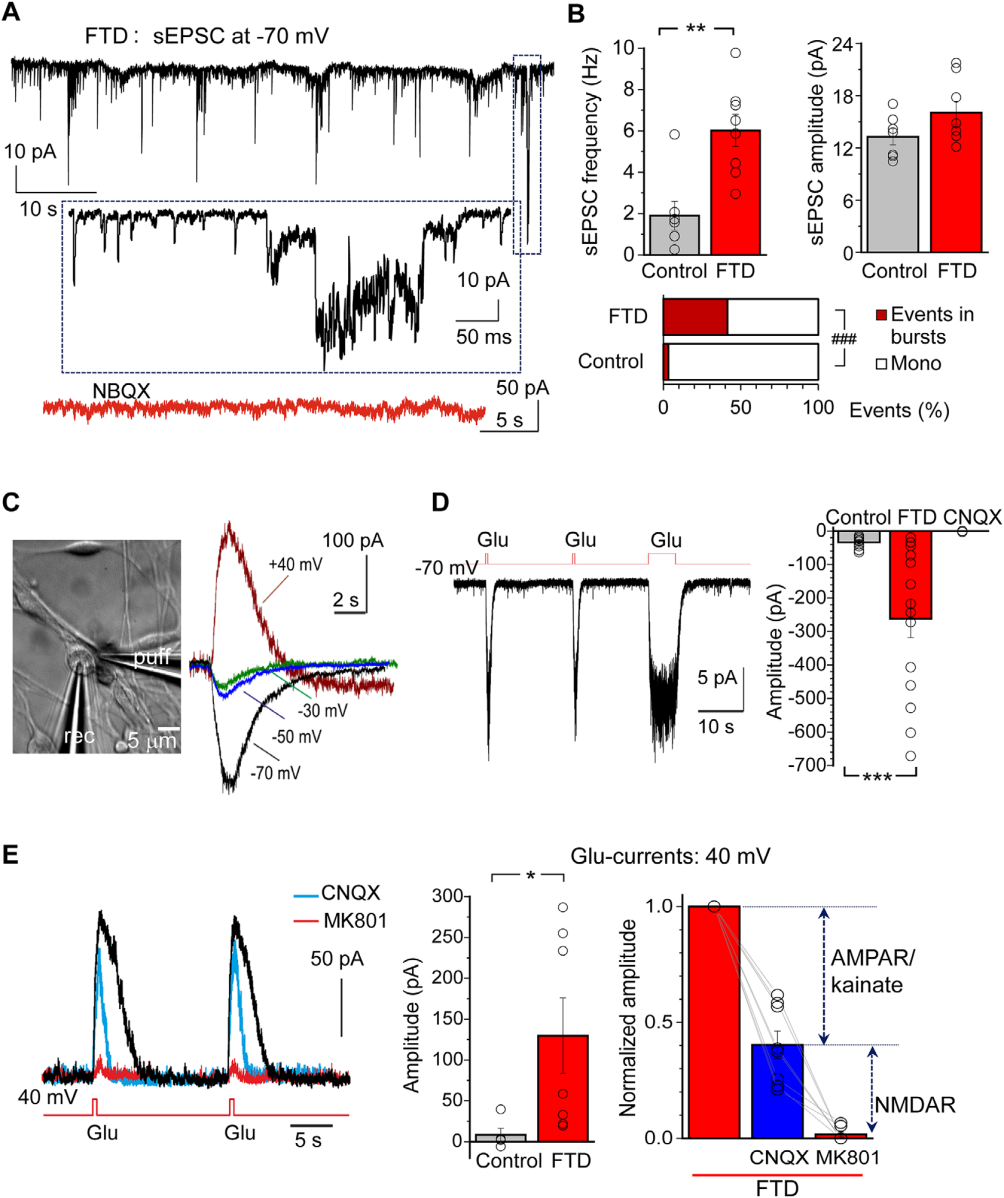
Pathological activity of frontotemporal dementia (FTD) neurons with the *MAPT* 10+16 mutation links to upregulated α‐amino‐3‐hydroxy‐5‐methyl‐4‐isoxazolepropionic acid receptor (AMPAR). A, Representative synaptic activity recording (spontaneous excitatory postsynaptic currents, sEPSC) made from FTD‐induced pluripotent stem cell (iPSC)‐derived neuron (≈150 DIV). Note irregular activity with bursts of sEPSC, which are solely AMPAR‐mediated (Vm = –70 mV) and completely abolished by 10 μM NBQX (lowest red trace). Box depicts an area illustrated on an expanded scale. B, Quantification of the sEPSC frequency (left), the current amplitude (right), and the proportion of bursts of synaptic events (bottom), relative to the total number of sEPSC recorded in control iPSC‐derived neurons (n = 1557 events, 7 neurons) and in FTD with the *MAPT* 10+16 mutation (n = 13683 events, 8 neurons). Data are median values. **P < 0.01 (Mann‐Whiney U‐test); ^###^ *P* < 0.001 (Fisher's exact test). C, Image, experimental arrangement as seen in the microscope (DIC channel) for local application of exogenous glutamate; recording (rec) and application (puff) pipettes are seen. Traces, glutamate‐evoked (100 μM, 500 ms) currents recorded from an IPSC‐derived neuron at different membrane potentials as noted. D, Left, example AMPAR‐mediated currents (Vm = –70 mV) in response to sequential glutamate applications (Glu, 100 μM) of different duration (500 ms and 5 s). Right, summary of the AMPAR‐mediated current amplitude in IPSC‐derived neurons from control lines (n = 11) and FTD (n = 10 neurons) at 150 DIV. Data are mean ± standard error of the mean (SEM). ***P < 0.001 (unpaired *t*‐test). E, Left, example glutamate‐evoked currents, recorded at 40 mV in a FTD iPSC‐derived neuron. Note, 10 μM CNQX reduced glutamate‐induced current at 40 mV (reflecting the AMPAR/kainate receptor activation) and 10 μM MK801 completely eliminated the current (representing the N‐methyl‐D‐aspartate receptor (NMDAR)‐mediated component). Right, statistics of the AMPAR/NMDAR‐mediated current amplitude (Vm = 40 mV) in IPSC‐derived neurons in control lines (n = 5 neurons) and FTD (n = 7 neurons), with quantification of the upregulated AMPAR and NMDAR in FTD neurons relative to the corresponding glutamate‐evoked current before antagonist applications. Data are mean ± SEM. *P < 0.05 (unpaired *t*‐test)

To evaluate synaptic dysfunction, we next recorded synaptic events (sEPSC), which revealed about two‐fold increase in the frequency of events occurred in neurons with the mutation (Figure 2B). These currents were AMPAR‐mediated because they were recorded at –70 mV and eliminated by the competitive AMPAR antagonist NBQX (Figure 2A). The increased frequency indicates boosted synaptic drive in FTD. Interestingly, the mutation did not change the sEPSC amplitude (*P* = 0.10; Figure 2B). Consistently, in 10+16 neurons, about ≈41% of all synaptic events occurred within high‐frequency bursts (Figure 2B), further confirming irregular, bursting activity in FTD.

To understand how neuronal dysfunction in FTD relates to changes in glutamate receptor functioning, we next recorded glutamate‐evoked currents. The currents were evoked by locally applied glutamate (see Methods) and recorded in human iPSC‐derived neurons at different membrane potentials (Figure 2C) in response to train of repeated glutamate puffs (500‐ms to 5‐s pulses; Figure 2D). In 10+16 iPSC‐derived neurons, the amplitude of glutamate‐evoked currents recorded at –70 mV was increased ≈7.6 times compared to that in control (Figure 2D). Because it was recorded at –70 mV and fully eliminated by a selective AMPA/kainate receptor antagonist CNQX, this indicates upregulated AMPAR in FTD neurons. The glutamate‐evoked current amplitude was also increased when recorded at +40 mV (above ≈4.4 times, Figure 2E). Such an increase in the current amplitude was reduced by CNQX (by ≈50%, *P* < 0.001 paired comparison), reflecting the AMPAR‐mediated component, while the subsequent application of an activity‐dependent NMDAR antagonist, MK‐801, fully eliminated the remaining current (Figure 2E). This indicates that in FTD human neurons with the *MAPT* mutation, the glutamate‐evoked current increase is due to upregulation of both AMPAR and NMDAR.

### 3.2 Increased AMPAR‐ and NMDAR‐mediated Ca^2+^ influx in iPSC‐derived neurons with the MAPT 10+16 mutation

**FIGURE 3:**
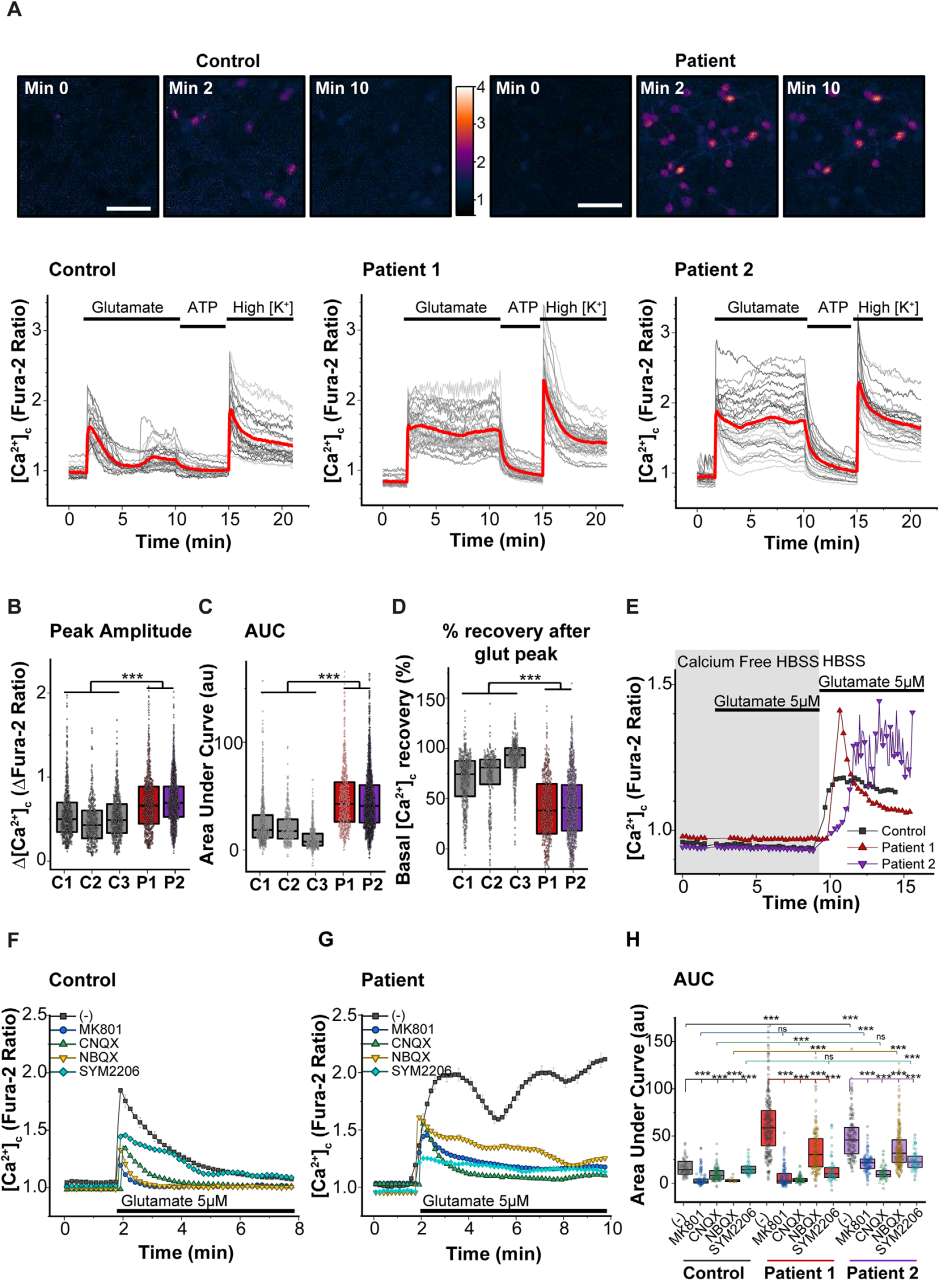
Glutamate‐induced calcium influx through α‐amino‐3‐hydroxy‐5‐methyl‐4‐isoxazolepropionic acid receptors (AMPARs) and N‐methyl‐D‐aspartate receptors (NMDARs) is upregulated in frontotemporal dementia (FTD)‐related induced pluripotent stem cell (iPSC)‐derived neurons with *MAPT* 10+16 mutation. A‐D, Cytosolic calcium levels ([Ca^2+^]_c_) of iPSC‐derived neurons from controls (C1‐3) and patients with the 10+16 mutation (P1‐2) in response to different stimulus were analyzed by live cell imaging using Fura‐2 AM. A, Color‐coded images and traces from a representative experiment. Images show Fura‐2 ratio levels at the beginning of the experiment (minute 0), in the peak after application of glutamate (minute 2), and at the end of the glutamate exposure (after 10 minutes) in control and patient. Scale bar: 25 μm. Representative traces show the calcium response of (C) control (n = 26), (D) patient 1 (n = 34), and (E) patient 2 (n = 29) neurons (red traces depicts the average) to the subsequent application of 5 μM glutamate, 100 μM ATP and 50 mM KCl, with media replacement before each application. B‐D, Peak amplitude (B), area under the curve (C), and percentage of basal calcium levels recovery after the peak at the end of the glutamate exposure (D; 100% represents a full recovery of the calcium basal levels, 0% represents the maintenance of the peak amplitude = no recovery) of the glutamate‐induced calcium response. Box plots represent the median, 25, and 75 percentiles; C1 n = 762, C2 n = 351, C3 n = 601, P1 n = 732, P2 n = 1364 neurons analyzed. Non‐parametric Kruskal‐Wallis H test, ***P < 0.0001. E, Representative traces of the cytosolic calcium levels of neurons (control n = 15, patient 1 n = 38, patient 2 n = 46). None of the cells showed a calcium signal after the application of 5 μM glutamate in calcium‐free HBSS (+ 0.5 mM ethylene glycol tetraacetic acid), discarding the role of metabotropic glutamate receptors. Reintroduction of glutamate in regular HBSS media (containing 2 mM CaCl_2_) reproduced the type of response observed in (A), indicating that ionotropic receptors are responsible for the altered signal. F‐H, Traces from a representative experiment showing the mean ± standard error of the calcium response of control (F) and patient (G) neurons to 5 μM glutamate after a pre‐treatment (20 minutes) with different ionotropic glutamate receptors antagonists. No drug pre‐treatment (–); NMDA antagonist MK801, 10 μM; AMPA/kainate antagonist CNQX, 20 μM; AMPA‐selective competitive antagonist NBQX, 20 μM; AMPA‐selective non‐competitive antagonist SYM2206, 10 μM. H, Area under the curve of the calcium signals. No drug pre‐treatment (–) (C, n = 49 neurons; P1, n = 250; P2, n = 152); MK801 (C, n = 125; P1, n = 128; P2, n = 108); CNQX (C, n = 128; P1, n = 119; P2, n = 80); NBQX, 20 μM (C, n = 17; P1, n = 175; P2, n = 233); SYM2206, 10 μM (C, n = 46; P1, n = 61; P2, n = 61). Box plots represent the median, 25, and 75 percentiles. Non‐parametric Kruskal‐Wallis H test was used to determine whether there were statistically significant differences between controls and patients, or between the treatments in each group (ns: non‐significant, ***P < 0.0001)

Impairment of glutamatergic signaling by AMPAR and NMDAR upregulation might disturb intracellular Ca^2+^ homeostasis, which is a known mechanism leading to neuronal death in tau‐induced FTD.^4^, ^5^ We therefore used live imaging to evaluate glutamate‐induced cytosolic Ca^2+^ signaling in the neurons. Application of a physiological concentration (5 μM) of glutamate induced a typical peak‐like elevation of [Ca^2+^]_c_ in control neurons, while the 10+16 *MAPT* mutation led to a calcium response of significantly higher amplitude and different shape (Figures 3A–3B). FTD neurons showed a sustained plateau of elevated [Ca^2+^]_c_ with most of them depicting a second (delayed) peak (Figure 3A). AUC was ≈two times larger; while recovery of basal [Ca^2+^]_c_ significantly reduced (≈to half) in FTD neurons (Figures 3C, 3D), and ultimately, was only achieved by washing out the agonist (Figure 3A). Basal [Ca^2+^]_c_ before stimulation was however similar between cohorts (Figure S2A in supporting information). Nevertheless, trains of spontaneous [Ca^2+^]_c_ rise were observed in some neurons from patient 2 (Figure 3A), in agreement with previous results.^5^ We also explored the calcium response to other stimuli that further helped us to distinguish neurons and glial cells (Figure 3A).^36^, ^37^ Application of ATP activates P2Y receptors and induces calcium signaling predominantly in glial cells, but not in mature neurons. In our preparations, there was only a small proportion of glial cells (typically ≈10%) that responded to ATP and displayed a glial‐like morphology, thus were not taken in further analysis. In addition, high [K^+^] medium was applied to depolarize neurons, leading to the opening of voltage gated calcium channels specifically in neurons. High [K^+^] induced a robust Ca^2+^ response with no significant difference between the groups (Figure S2B).

Metabotropic glutamate receptors (mGlu) can contribute to the [Ca^2+^]_c_ transients by releasing Ca^2+^ from the endoplasmic reticulum. Application of 5 μM glutamate in the absence of extracellular Ca^2+^ did not induce any changes in [Ca^2+^]_c_ (Figure 3E), discarding the role of mGlu, and confirming 10 + 16 mutation in *MAPT* alters calcium signaling through ionotropic glutamate receptors.

Augmented Ca^2+^ influx was indeed blocked by NMDAR and AMPAR antagonists, such as MK‐801, CNQX, NBQX, and SYM2206 (Figures 3F–3H). Each of those significantly reduced the AUC and the peak [Ca^2+^]_c_ amplitude, to a varied extent (Figure S2C). Importantly, neurons with the mutation recovered basal [Ca^2+^]_c_ in the presence of AMPAR/NMDAR antagonists (Figure S2D), discarding an impairment in Ca^2+^ efflux in the mechanism of glutamate‐induced Ca^2+^ overload in FTD.

To further confirm the increase in AMPAR/NMDAR‐mediated Ca^2+^ permeability, we stimulated the cells with the selective agonists. Bath application of NMDA (20 μM, in Mg^2+^‐free medium) or AMPA (20 μM) to neuronal cultures evoked a [Ca^2+^]_c_ rise, which was, again, of a higher amplitude, with a larger AUC, in neurons with the 10+16 *MAPT* mutation compared to healthy controls (Figures S3A, S3B, S3D, S3E). Interestingly, application of kainate induced a higher rise in the patients, but [Ca^2+^]_c_ at the end of the experiment was not significantly different between all the controls and patients (Figures S2C, S2F). These data point to the tau‐induced upregulation of Ca^2+^‐permeable AMPAR and NMDAR in FTD neurons.

### 3.3 Introduction of the 10+16 MAPT mutation impairs calcium signaling and mitochondrial function in genetically engineered neurons

**FIGURE 4:**
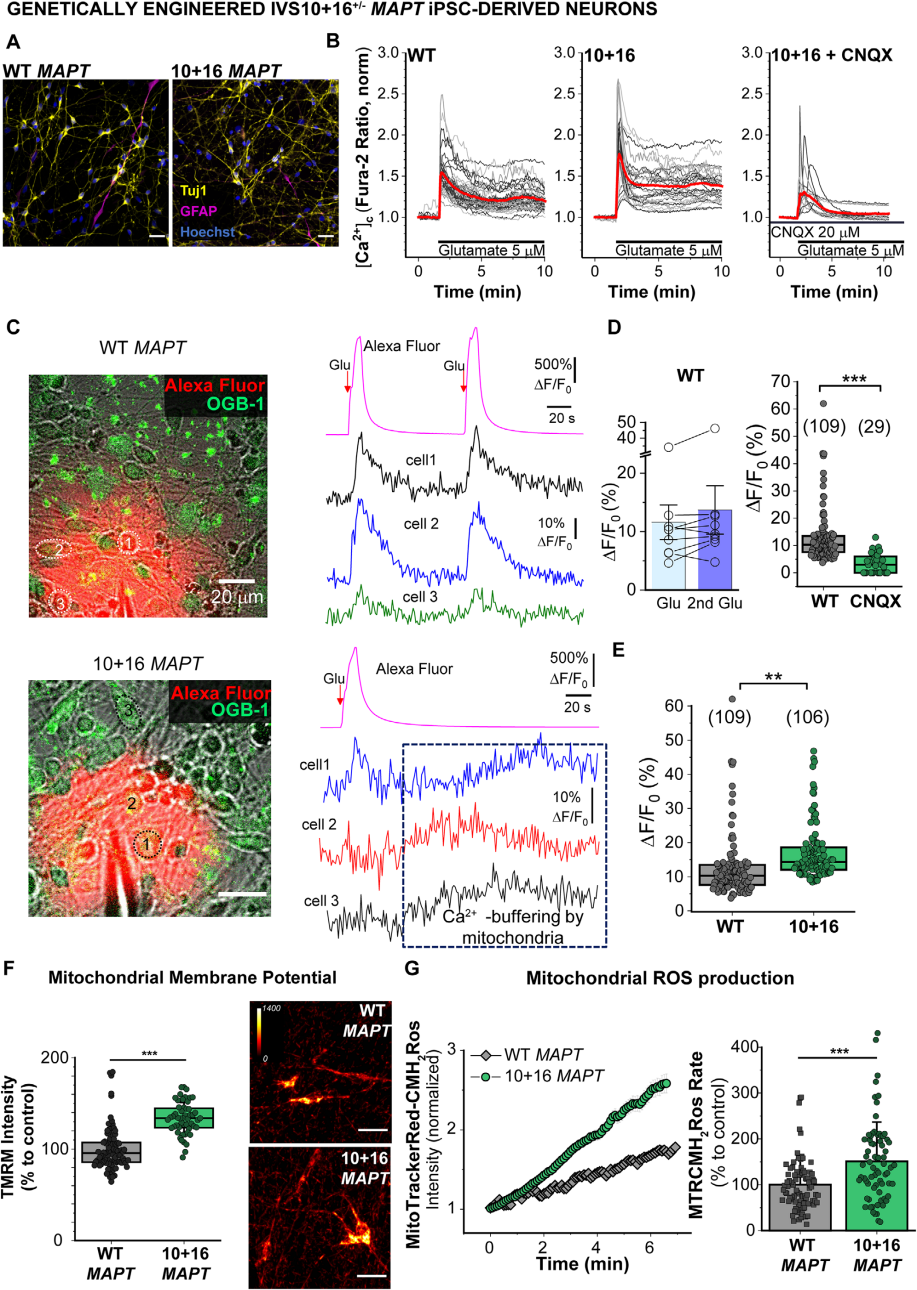
Genetically engineered 10+16 *MAPT* induced pluripotent stem cell (iPSC) neurons display increased glutamate‐induced calcium influx and mitochondrial reactive oxyten species (ROS) compared to isogenic wild‐type (WT) *MAPT* controls. A, Immunostaining of the genetically engineered 10+16 *MAPT* iPSC‐neurons and their isogenic WT *MAPT* control at D125, showing the neuronal marker Tuj1, the astrocytic marker GFAP and the nuclear dye Hoechst. Scale bar: 20 μm. B, Traces from one representative experiment showing the calcium response (measured by Fura‐2 ratio) to 5 μM glutamate in n = 44 wt *MAPT* neurons; n = 29 10+16 *MAPT* neurons and n = 14 10+16 *MAPT* neurons pre‐treated for 20 minutes with the AMPA/kainate antagonist CNQX 20 μM. C‐E, Two‐photon excitation (2PE) imaging of the intracellular Ca^2+^ dynamics (OBG‐1 signal) in wt and 10+16 *MAPT* lines. C, Images show experimental arrangement for focal application of glutamate (10 μM) through a micropipette positioned in close proximity of the cells; combined transmitted light, OGB‐1 (green), and Alexa Fluor‐594 (red) channels during glutamate puff; *λ* ^2P^_ex_ = 800 nm in WT neurons (top image) and 10+16 *MAPT* neurons (bottom image). Traces, time course of the Ca^2+^ rise over indicated regions of interest (ROI, dotted circles on images) before and after repetitive glutamate puffs; top trace, Alexa Fluor‐594 diffusion profile across the field of view; red arrow, timing of glutamate puff. Note extended Ca^2+^‐signals induced by a single glutamate puff in 10+16 *MAPT* neurons, suggesting mitochondrial involvement. D, Quantification of the glutamate‐induced Ca^2+^‐rise (OGB‐1 fluorescence intensity ratio) in individual neurons in wt tau neurons, eliminated by CNQX (right). E, Statistical comparison of the glutamate‐induced Ca^2+^‐rise in control (WT) and 10+16 *MAPT* groups. Data are mean ± standard error of the mean (SEM). ***P < 0.001; **P < 0.01 (Mann‐Whiney U‐test). Number of analyzed neurons indicated. F, Mitochondrial membrane potential assessed by TMRM staining. Box plots represent the median, 25 and 75 percentiles; WT, n = 93 neurons; 10+16, n = 52. Non parametric Mann Whitney test, ***P < 0.0001. Color‐coded representative images. Scale bar: 20 μm. G, Mitochondrial ROS production assessed with MitoTrackerRed‐CMH_2_Ros live imaging. Traces from one representative experiment (left) and rate of mito ROS production in independent neurons. Histograms represent the average±SEM of WT *MAPT*, n = 87 neurons and 10 + 16 *MAPT* n = 72. Two‐sample *t*‐test, ***P < 0.0001

To confirm 10+16 *MAPT* mutation was indeed causing the observed calcium impairment in patients, we additionally used neurons derived from a genetically engineered iPSC line, in which the 10+16T monoallelic mutation in *MAPT* was introduced into a control iPSC line.^27^ Immunocytochemistry validated positive neuronal staining with Tuj1 (Figure 4A), with some cells positively stained with the astrocytic marker GFAP (up to ≈10%), confirming a pattern similar to that in our FTD‐iPSC lines.^24^

**FIGURE 5:**
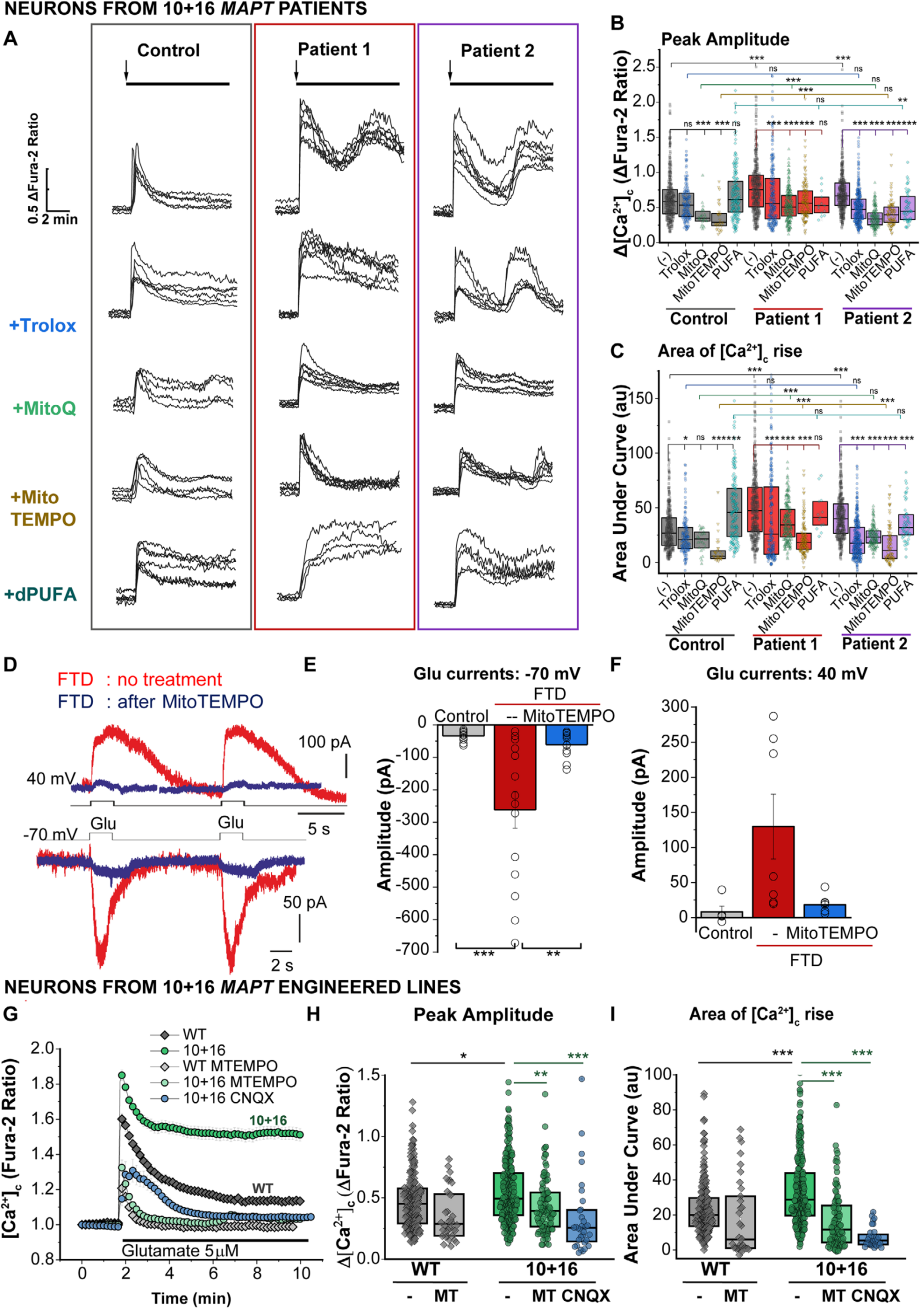
Mitochondrially located antioxidants reduce the upregulated glutamate‐induced calcium signals in *MAPT* 10+16 induced pluripotent stem cell (iPSC)‐derived neurons. A, Representative traces showing the calcium response to 5 μM glutamate (application indicated by the arrow), after pre‐treatment of control, patient 1 or patient 2 neurons with different antioxidants: no drug pretreatment (–), water‐soluble analogue of vitamin E, Trolox (100 μM, 2 hours); mitochondrially located antioxidants MitoQ and MitoTEMPO (100 nM, 1 hour) or inhibitor of lipid peroxidation, d‐PUFA (10 μM, 48 hours). B‐C, Peak amplitude (B) and area under the curve (C) of the calcium response induced by glutamate, in the presence or absence of the different antioxidants. No drug (C, n = 346 neurons; P1, n = 393; P2, n = 405); Trolox (C, n = 154; P1, n = 218; P2, n = 336); MitoQ (C, n = 26; P1, n = 218; P2, n = 154); MitoTEMPO (C, n = 33; P1, n = 140; P2, n = 103); d‐PUFA (C, n = 161; P1, n = 13; P2, n = 55). Box plots represent the median, 25‐ and 75 percentiles. Non‐parametric Kruskal‐Wallis H test was used to determine whether there were statistically significant differences between controls and patients, or between the treatments in each group (ns: non‐significant, *P < 0.05, ** *P* < 0.01, *** *P* < 0.0001). D‐F, Patch‐clamp recordings of the glutamate‐evoked currents in iPSC‐derived neurons. D, Representative whole‐cell recordings in FTD‐iPSC‐derived neurons with the *MAPT* 10+16 mutation without treatment (red) and post‐treatment with MitoTEMPO (2 hours pre‐incubation) at –70 mV (lower traces) and 40 mV (upper traces) in response to local glutamate (Glu) puffs (as indicated). Statistical comparison of the glutamate‐evoked current amplitudes at –70 mV (E) and 40 mV (F) between control (gray) and FTD groups without treatment (red) and treated with mitochondrial antioxidant (blue). Data are mean ± standard error of the mean (SEM). *** *P* < 0.001; **P < 0.01 (unpaired *t*‐test). G‐I, Changes in [Ca^2+^]_c_ in response to glutamate after the pretreatment of the genetically engineered 10+16 *MAPT* neurons and isogenic WT *MAPT* control with the mitochondrial antioxidant MitoTEMPO (MT, 100 nM, 1 hour) or the AMPA/kainate antagonist CNQX (20 μM, 20 minutes). G, Traces represent the average±SEM from one representative experiment, of WT, n = 45; WT+MT, n = 8; 10+16. n = 36; 10+16+MT, n = 21; 10+16+CNQX, n = 14 neurons. Quantification of the peak amplitude (H) and area under the curve (I) in individual neurons. Box plots represent the median, 25 and 75 percentiles; WT, n = 222 neurons; WT+MT, n = 34; 10+16, n = 279; 10+16+MT, n = 97; 10+16+CNQX, n = 32. Non‐parametric Kruskal‐Wallis test, * *P* < 0.05, ** *P* < 0.01, *** *P* < 0.0001)

Alike FTD‐neurons, glutamate‐induced [Ca^2+^]_c_ rise in genetically engineered 10+16 iPSC‐derived neurons presented a higher peak amplitude, larger AUC, and slower [Ca^2+^]_c_ recovery compared to their isogenic wt tau control, that were reverted with the AMPA/kainate antagonist CNQX (Figure 4B, Figures 5G–5I, Figures S5B, S5C in supporting information). This was further confirmed by 2‐photon excitation (2PE) imaging (see Methods). Time‐lapse imaging (≈1 s per frame) demonstrated a robust transient [Ca^2+^]_c_ rise in wt‐tau neurons in response to repetitive localized applications of glutamate (10 μM), whose kinetics well resembled a spatial‐temporal profile of agonist application/diffusion (a few seconds time‐course; Figure 4C). Glutamate‐evoked [Ca^2+^]_c_ transients were eliminated by CNQX (Figure 4D). In genetically engineered 10+16 *MAPT* neurons, the [Ca^2+^]_c_ rise had a dramatically slower decay and a higher peak amplitude compared to the isogenic wt tau cells (Figures 4C, 4E). Electrophysiological recordings also demonstrated increased glutamate‐induced currents in 10+16 *MAPT* neurons, which were eliminated by CNQX at –70 mV (Figure S4A in supporting information) and CNQX with APV at 40 mV (Figure S4B).

Importantly, genetically engineered 10+16 neurons also mimicked the mitochondrial dysfunction we previously described in 10+16 FTD neurons.^8^ Engineered 10+16 neurons exhibited a hyperpolarized mitochondria and an increased rate of cytosolic and mitochondrial ROS production (Figures 4F–4G, Figures S4C, S4D) compared to their isogenic wt tau neurons. Depolarization of the mitochondria with rotenone reduced ROS production in 10+16 neurons (Figures S4C, S4D), indicating mitochondrial hyperpolarization was the underlying cause of the elevated ROS, as previously observed in patients’ neurons.^8^ Thus, both calcium‐deregulation and mitochondrial dysfunction appear to be specifically mediated by this tau mutation.

### 3.4 Mitochondrially located antioxidants prevent the glutamate‐induced calcium deregulation in MAPT 10+16 iPSC‐neurons

Indeed, we previously showed that 10+16 FTD‐neurons are more vulnerable to calcium‐induced cell death^5^ and that mitochondrial ROS overproduction is a key pathological event, leading to neuronal death that could be prevented with mitochondrially targeted antioxidants.^8^, ^23^ We therefore reasoned whether the neuroprotective effect of mitochondrial (and other) antioxidants could be exerted by targeting AMPAR and NMDAR‐mediated calcium overload. We implemented several treatments: water soluble analogue of vitamin E, Trolox; mitochondrially located antioxidants MitoQ and MitoTEMPO; and deuterated PUFAs (non‐antioxidant compounds which inhibit lipid peroxidation^38^; Figure 5A). Trolox treatment reduced the peak amplitude of glutamate‐evoked [Ca^2+^]_c_ rise in patients’ neurons with the 10+16 mutation, improved the AUC, and the recovery of basal [Ca^2+^]_c_ (Figures 5A–5C, Figure S5A). In contrast, treatment with D‐PUFAs did not significantly changed peak amplitude or AUC, nor facilitated recovery of basal [Ca^2+^]_c_ in the patients (Figures 5A–5C, Figure S5A) suggesting no considerable role of lipid peroxidation in the glutamatergic impairment induced by tau. However, treatment with mitochondrial antioxidants MitoQ and MitoTEMPO restored the augmented glutamate‐induced [Ca^2+^]_c_ rise in neurons with the *MAPT* 10+16 mutation, to the full extent (Figures 5A–5C, Figure S5A). Interestingly, both MitoQ and MitoTEMPO improved [Ca^2+^]_c_ handling in the control cohort (Figures 5A–5C, Figure S5A). Mitochondrial antioxidants produced a greater effect than the general antioxidant trolox on recovering the impaired [Ca^2+^]_c_ signaling in FTD neurons, thus emphasizing the key impact of mitochondrial ROS in the mechanism of tau‐induced AMPAR and NMDAR upregulation. Indeed, MitoTEMPO also restored the augmented glutamate‐evoked [Ca^2+^]_c_ rise in genetically engineered 10+16 neurons, reducing peak amplitude, AUC, and enhancing recovery of basal [Ca^2+^]_c_ (Figures 5G–5I, Figure S5C).

Whole‐cell recordings further confirmed that mitochondrial antioxidants restored the AMPAR‐mediated currents (recorded at –70 mV and eliminated by CNQX) in FTD neurons to a level of those in healthy control lines (Figures 5D–5E). Similarly, the amplitude of glutamate‐evoked currents recorded at 40 mV decreased after MitoTEMPO treatment in neurons with the mutation (Figures 5D, 5F).

Thus, tau‐induced mitochondrial ROS overproduction is the underlying cause of NMDAR‐ and AMPAR‐mediated calcium deregulation in both FTD and engineered 10+16 neurons.

### 3.5 Mitochondrial antioxidants reduce the surface levels of specific AMPAR and NMDAR subunits elevated in patients’ neurons

**FIGURE 6:**
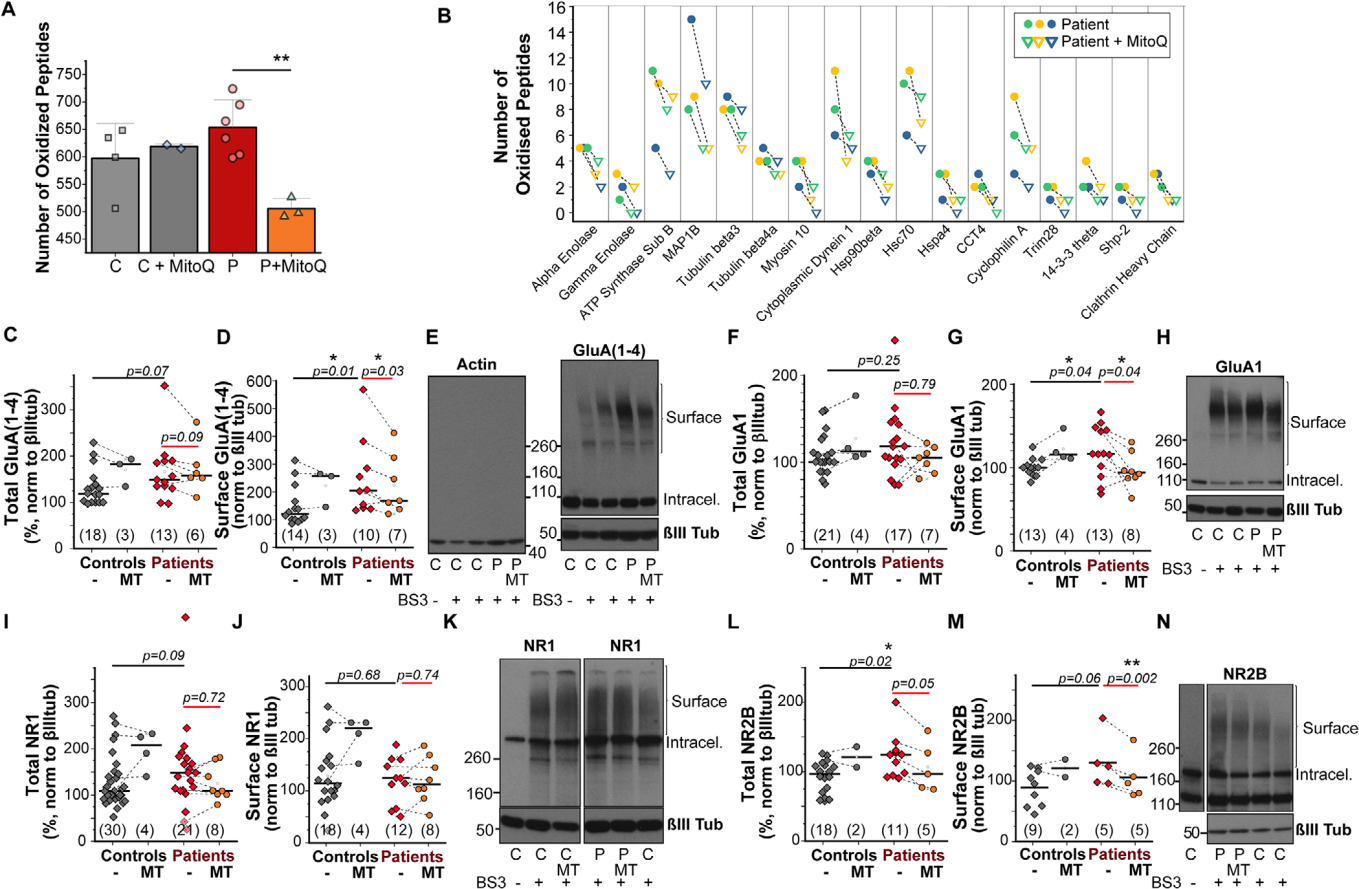
Mitochondrial antioxidants reduce the surface levels of specific α‐amino‐3‐hydroxy‐5‐methyl‐4‐isoxazolepropionic acid receptor (AMPAR) and N‐methyl‐D‐aspartate receptor (NMDAR) subunits elevated in patients’ neurons. A,B, Redox proteomics analysis of oxidized peptides in patients (P) and control (C) groups with and without MitoQ treatment (100 nM). A, Histograms represent the mean ± standard deviation of the number of oxidized peptides in control n = 4, c+mitoQ n = 2, patients n = 6, patients+mitoQ, n = 3 preparations. ** *P* < 0.01, two‐way analysis of variance. B, Specific proteins showing a reduction in the number of oxidized peptides after the treatment with MitoQ (100 nM) in patients as analyzed by redox proteomics. Only proteins that showed a reduction in the three samples investigated are shown. C‐N, Intracellular and surface distribution of different AMPAR and NMDAR subunits analyzed by western blot after the crosslinking of the superficial receptors using BS^3^. E, H, K, N, Representative western blots showing the bands corresponding to the intracellular and surface (crosslinked) subunits of: (E) all the AMPAR subunits GluA(1‐4), detected with a pan‐AMPA antibody, (H) AMPAR subunit GluA1, (K) NMDAR subunit NR1, (N) NMDAR subunit NR2B. Neuron‐specific βIII tubulin was used as a loading control. Absence of higher molecular weight bands in the actin band (E) confirms that intracellular proteins were not crosslinked. Histograms show the quantification of the number of samples indicated in brackets, lines represent the median. For the quantification of the total levels, intracellular and surface levels were added. In the total levels group, some additional experiments using non‐crosslinked samples were also included. In all cases, data was normalized to control for each experiment. Statistical significance between control and patients was analyzed with the non‐parametric Mann‐Whitney test in all cases except (G), which followed a normal distribution and two‐sample t‐test was used, number of samples analyzed is indicated in brackets. Specific samples, as indicated by the dotted lines, were treated with the mitochondrial antioxidant MitoTEMPO (MT, 100 nM, 1h). Statistical significance of the effect of MitoTEMPO was analyzed with the non‐parametric paired Wilcoxon signed‐rank test, except in (G) where the paired *t*‐test was used. Number of samples analyzed is indicated in the MT column

Excessive ROS production might exert a pathogenic role by mediating the oxidation of numerous proteins and altering their structure, hence function.^37^, ^39^ Some amino acid oxidations can be reversed (i.e., methionine or cysteine oxidation), while amino acid carbonylation remains irreversible. Accumulation of reversibly oxidized amino acids may act as a regulatory switch.^40^, ^41^ To further understand the role of mitochondrial ROS overproduction in FTD pathogenesis we next explored the pattern of protein oxidation in neurons using redox proteomics. As expected, we observed an increase in the amount of oxidized peptides in patients compared to control (Figure 6A). Pre‐incubation of neurons with MitoQ effectively reduced the number of oxidized peptides (by > 20%) in FTD samples (Figure 6A) and shed some light on potential protein candidates to explain how mitochondrial ROS mediates tau pathology and glutamatergic dysfunction (Figure 6B). Notably, MitoQ reversed the oxidation of proteins such as MAP1B, clathrin, or Hsc70, known to regulate AMPAR and NMDAR trafficking, so we next explored this possibility by analyzing the distribution of these receptors between cytosol and plasma membrane of neurons using a crosslinking assay as described in Methods.

AMPARs are tetramers composed of different subunits (GluA1‐4), with GluA1 and GluA2 predominant, and the lack of editing of GluA2 conferring the receptor calcium permeability. There was no significant difference in the total levels of all four AMPAR subunits (GluA1‐4) measured with a pan AMPAR antibody (Figure 6C), or specifically GluA1 (Figure 6F) between control and FTD neurons. However, their surface expression was significantly higher in patients (Figures 6D, 6E, 6G, 6H). Importantly, treatment of FTD neurons with the mitochondrial antioxidant MitoTEMPO decreased the surface level of GluA1 and GluA(1‐4) (Figures 6D, 6E, 6G, 6H), with no significant effect on total subunit content (Figures 6C, 6F). This indicates increased mitochondrial ROS contributes to the trafficking of AMPARs, and specifically the calcium‐permeable subunit GluA1, to the cellular membrane in patients.

NMDARs are also tetramers composed of two obligatory NR1, plus NR2(A‐D) (or more rarely NR3) subunits, which confer the receptor‐specific signaling properties. NR1 total and surface levels were similar between control and FTD neurons, and were unaffected by mitoTEMPO (Figures 6I–6K). However, NR2B, known to predominately locate in extrasynaptic membranes and contribute to excitotoxicity,^42^ was highly expressed in patients (Figure 6L), and MitoTEMPO significantly reduced its presence in the cell membrane (Figures 6M, 6N). These results indicate that although membrane expression of NMDARs is similar as indicated by NR1, specific NR2B‐containing receptors involved in excitotoxicity are upregulated in the patients and can be modulated by mitochondrial antioxidants, providing a mechanistic basis of the neuroprotective action of these compounds.

### 3.6 Extracellular 4R tau impairs the glutamate‐induced calcium response of control neurons by increasing mitochondrial ROS production

**FIGURE 7:**
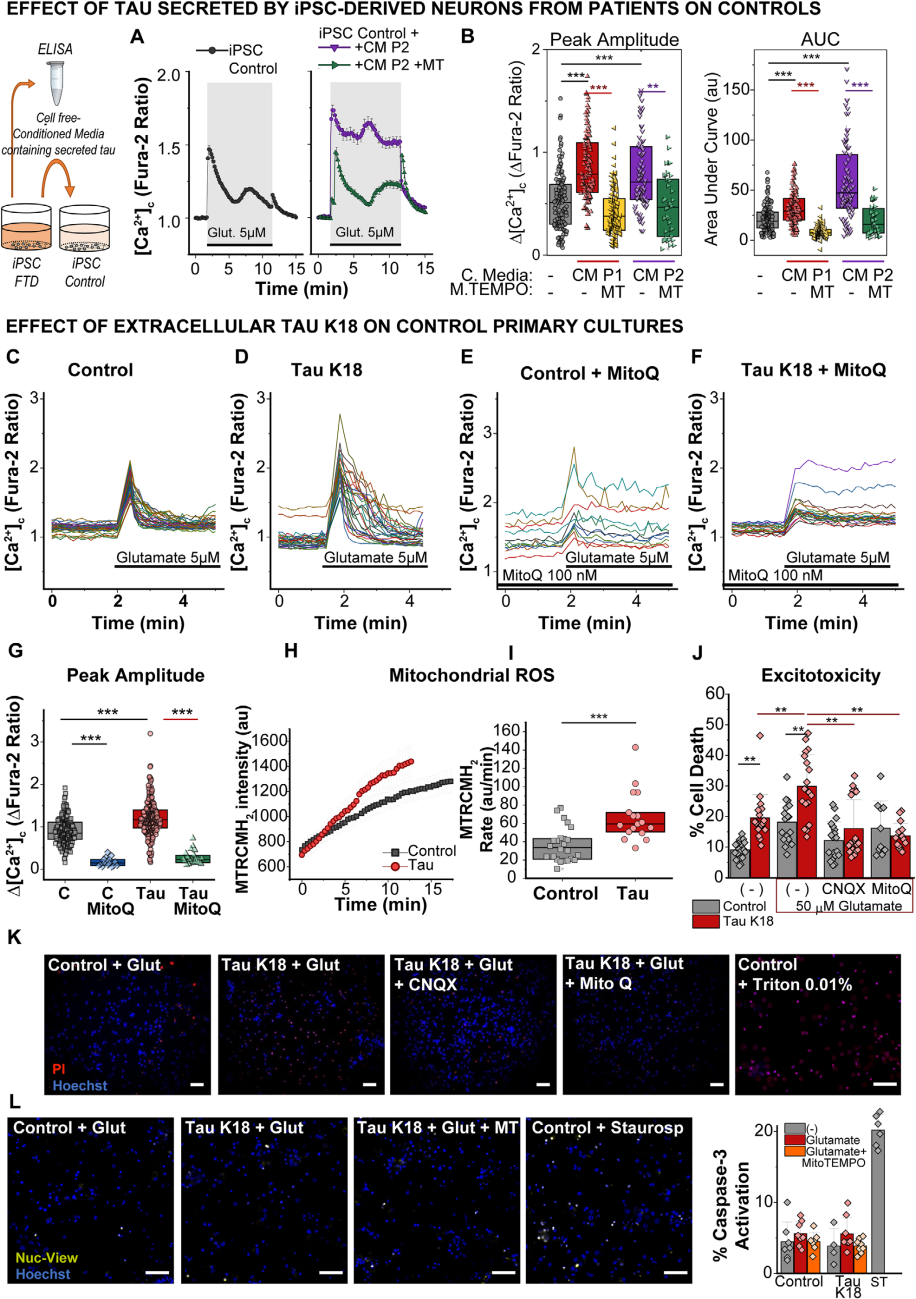
Extracellular tau alters the glutamate‐induced Ca^2+^ response of induced pluripotent stem cell (iPSC) and primary control neurons by increasing mitochondrial ROS production. A, B, Conditioned media (CM) containing secreted tau from each of the frontotemporal dementia (FTD)‐related *MAPT* 10+16 iPSC‐ patients neurons (P1 and P2) was applied to control iPSC‐neurons and incubated for 48 hours. Calcium responses to glutamate were studied afterward in the control, with or without pre‐treatment with the mitochondrial antioxidant mitoTEMPO (MT, 100 nM, 1 h). A, Traces from a representative experiment. B, Quantification of the peak amplitude and area under the curve in individual neurons. Box plots represent the median, 25 and 75 percentiles; C, n = 135 neurons; C+CM P1, n = 112; C+CM P1 + MT, n = 120; C+CM P2, n = 84; C+CM P2 + MT, n = 38. Non‐parametric Kruskal‐Wallis test, **P < 0.01, *** *P* < 0.0001. C‐F, Representative traces illustrating the glutamate‐induced calcium response on rat primary neurons to a physiological (5 μM) concentration of glutamate in control conditions (C), after the incubation of the cells for 24 hours with 300 nM K18 tau (D), and after preincubating the cells for 1 hour with 100 nM MitoQ prior to the experiment (E, F). G, Amplitude of the calcium peak in the conditions described before. Box plots represent the median, 25 and 75 percentiles; C, n = 181 neurons; C + MitoQ, n = 17; Tau, n = 234; Tau+MitoQ, n = 23. Non‐parametric Kruskal‐Wallis test, ***P < 0.0001). H‐I, Rate of mitochondrial reactive oxygen species (ROS) production was assessed with MitoTrackerRedCMH_2_Ros in control primary neurons treated or not with 300 nM K18 tau for 24 hours. Representative traces from one experiment (H) and quantification of the rate of ROS production (I) in control (n = 24 neurons) and tau‐treated (n = 18) neurons. Box plots represent the median, 25, and 75 percentiles. Non parametric Mann‐Whitney test, ***P < 0.0001. J, Percentage of cell death in basal conditions (control n = 19 fields analyzed; tau n = 19), or 24 hours after a short treatment (30 minutes) with 50 μM glutamate in the absence (C, n = 18; tau n = 19); or presence of the inhibitor of mitochondrial ROS, MitoQ 100 nM, (C, n = 10; tau, n = 15) or the AMPA/kainate receptor antagonist, CNQX 20 μM (C, n = 19; tau, n = 19). Histograms represent the mean ± standard deviation, two‐way analysis of variance with Bonferroni post‐hoc test, **P < 0.01. K, Representative images of cell death experiments: propidium iodide (red fluorescence) labels dead cells, and Hoechst (blue fluorescence) labels all cells. Triton was used as a positive control. Scale bar: 50 μm. L, Representative images showing caspase‐3 activation in individual cells as bright NucView488 nuclei colocalizing with the nuclear marker Hoechst. Staurosporine (ST)1 μM for 3 hours was used as a positive control. Right panel, percentage of caspase‐3 positive cells. Scale bar: 50 μm

In the recent years, increasing evidence supports that tau spreads through the brain in a “prion‐like” manner, in a mechanism involving extracellular tau release and uptake by cells.^43^, ^44^ We found that iPSC‐derived neurons were able to secrete tau and interestingly, tau secretion in patient 2 was significantly higher than in control (Figure S6B in supporting information). Notably, this patient also showed spontaneous calcium oscillations in the absence of any stimulation (Figure 3A), which is consistent with other authors showing that increased neuronal activity stimulates the release of tau.^45^ We then hypothesized if secreted extracellular tau was also able to alter the glutamate‐induced calcium signaling of the cells. To this end, we treated the three control lines either with their own supernatant or with the supernatant obtained from the two 10+16 FTD iPSC‐lines for 48 hours (Figure 7). Control cells treated with conditioned media from patients depicted a similar alteration in the glutamate‐induced calcium signaling as patients’ cells themselves, including increased peak amplitude, AUC, and sustained shape of the [Ca^2+^]_c_ transients (Figures 7A, 7B; Figures S6C–S6F). Importantly, pretreatment of control neurons with the mitochondrial antioxidant mitoTEMPO again significantly restored the altered response induced by the conditioned media from mutant cells (Figures 7A, 7B). Total tau concentration in the conditioned media used was similar in control and patients (Figure S6A), but only the supernatant from patients induced an alteration in the glutamate‐induced calcium response. At the age our iPSC‐neurons were used for experiments (120–150 DIV), 10+16 neurons show a robust expression of exon 10, and therefore 4R tau production, whereas only low levels of 4R tau are observed in controls.^26^ This suggests a specific role for 4R tau isoform, whose production is enhanced by the 10+16 mutation, in the mechanism of action. To confirm this point, we used K18 tau, which is a construct from wt 4R tau comprising the four‐repeat region of the protein, to treat primary rat cortical neurons (300 nM K18 tau, 24 h). Similarly to human iPSC‐derived neurons, tau‐treated primary neurons also showed upregulated calcium responses to a physiological concentration of glutamate (5 μM), which were restored with the mitochondrial antioxidant MitoQ (Figures 7C–7G). Notably, mitochondrial ROS production was also increased by K18 tau (Figures 7H, 7I).

Calcium deregulation in tau‐treated primary neurons became even clearer under exposure of these cells to toxic concentrations of glutamate. Thus, 50 μM glutamate induced the initial peak and delayed calcium deregulation typical for this concentration, which was higher in tau‐treated cells (Figures S6G, S6H, S6K). And again, the mitochondrial antioxidant MitoQ significantly decreased the calcium signal in response to 50 μM glutamate in both control and tau‐treated neurons (Figures S6I–S6K). The specific role of 4R tau was further confirmed by treating primary neurons with extracellular 3R tau. Interestingly, 3R tau also induced an altered calcium response in the neurons but by a different mechanism than 4R tau, because it was not modulated by mitochondrial ROS (Figure S6L), which indeed were only upregulated by 4R and not 3R tau (Figure S6M). Taken together, these results confirm that extracellular or secreted 4R tau recapitulate the pathological phenotype of 10+16 mutation.

### 3.7 Tau‐enhanced excitotoxicity can be reduced by mitochondrial antioxidants

Treatment of primary cortical neurons with K18 tau significantly increased neuronal death (Figures 7J–7K) as previously observed in human iPSC‐derived neurons with the 10+16 mutation.^8^ Caspase‐3 activation was similar among all the conditions (Figure 7L) suggesting necrosis was the preferential cell death mechanism. Importantly, neuronal death was also significantly higher in tau‐treated cells after 24 hours of an (excitotoxic) 30‐minute exposure to 50 μM glutamate (Figures 7J–7K). Mitochondrial antioxidant MitoQ significantly prevented excitotoxicity in a similar way as the glutamate ionotropic receptor antagonist CNQX (Figures 7J–7K). These results highlight the key role of mitochondrial ROS in tau‐induced glutamatergic dysfunction and the neuroprotective effect of these compounds in calcium‐induced cell death in FTD.

## 4 DISCUSSION

Here we report a direct link among mitochondrial ROS, calcium signaling, and glutamatergic transmission deregulation, which might lead to early dysfunction preceding neuronal loss in tauopathies, and, according to our results, is also involved in the mechanism of neurodegeneration.

Our data demonstrate that in 10+16 *MAPT* human neurons, NMDA‐ and AMPA‐mediated signaling is upregulated, as shown by the enhanced calcium responses induced by physiological concentrations of glutamate and the upregulated conductance and firing activity observed in the electrophysiology experiments. In agreement with our results, it was previously published that tau‐mediated NMDA receptor impairment underlies the dysfunction of a selectively vulnerable network in a mouse model of FTD,^46^ the enhanced effect of mutated tau on excitotoxicity via NMDA,^47^ and the involvement of calcium dysregulation in the mechanism of cell death of FTD iPSC‐neurons with different *MAPT* mutations.^4^ Interestingly, 10+16 *MAPT* mutation in our experiments also induced AMPA receptors deregulation similarly to FTD‐associated mutant CHMP2B in mice, where AMPA deregulation led to social behavioral impairments.^48^ A number of studies show altered expression of glutamate receptors in FTD and tau‐related diseases.^49^, ^50^, ^51^ Our data strongly suggest that the glutamatergic deregulation is reversible, and more sensitive to mitochondrial antioxidants compared to general ROS scavengers. We show that the surface expression of AMPAR and NMDAR is increased in 10+16 neurons and can be restored with mitochondrial antioxidants. This was not a general effect for all the AMPAR and NMDARs, as NR1 surface levels and therefore, general NMDAR surface levels, were not altered. Instead, specific subunits trafficking such as the calcium permeable GluA1 and NR2B were modified by tau‐induced mitochondrial ROS, although the role of other subunits cannot be discarded. Interestingly, GluN2B‐containing NMDA receptors are preferentially found in the extrasynapses and its activation is more linked to excitotoxicity. Indeed, several studies support the role of tau in the modulation of these receptors.^52^, ^53^, ^54^ One of the most important findings in our study is that tau mediates this modulation through mitochondrial ROS: we provide evidence of a direct link between mitochondrial dysfunction and glutamatergic transmission impairment. Most of the studies in this field reported the effect of glutamate receptor activation in physiology and pathology (excitotoxicity) on mitochondrial membrane potential, mitochondrial calcium, and ATP^55^, ^56^ for review. However, how mitochondria modify the glutamatergic signal in physiology, and mitochondrial dysfunction in particular, is poorly understood. That kind of effect of mitochondrial ROS on AMPA and NMDA receptors is shown for the first time to our knowledge. Considering the protective effect of mitochondrially located antioxidants, it should be mediated by ROS produced in the mitochondrial matrix. The lifetime of superoxide anion, which is initially produced in the ETC, is in the nanosecond range^22^ and it should be converted to hydrogen peroxide and transported to other parts of the cell.

It was confirmed by redox proteomics data that MitoQ protects mitochondrial and cytosolic proteins against oxidation in the iPSC patient neurons. Considering the redox sensitivity of the glutamate receptors (shown by the activation of NADPH oxidases^57^) the effect of mitochondrial ROS on the glutamate‐induced calcium signalling could be used in the physiological regulation of AMPA and NMDA conductivity. Among the oxidized proteins detected in the redox proteomics analysis to be reversed by MitoQ treatment in the patients, we found proteins implicated in glucose metabolism, like the beta subunit of the mitochondrial ATP synthase; and the gamma and alpha subunits of the glycolytic enzyme enolase—the latter has been consistently reported to be oxidatively modified in AD patients’ brains and animal models of the disease.^58^, ^59^, ^60^ Oxidative modifications of metabolism‐related enzymes have been linked to a diminished enzymatic function^37^ and contribute to the altered glucose metabolism occurring in neurodegenerative disorders, which might also affect the synaptic function, due to its high energy requirements. We previously showed glucose metabolism was altered in 10+16 neurons,^8^ which were able to maintain ATP levels at the age they were studied by different compensatory mechanisms. This chronic impairment in the energy supply might be the trigger for neuronal dysfunction and neurodegeneration. Indeed, we demonstrate that mitochondrial bioenergetics dysfunction, shown by the increased mitochondrial membrane potential, is a key event in the pathology, as it drives the increased mitochondrial ROS production.

Proteomics data suggest that the protective effect of MitoQ is related to the regulation of AMPAR and NMDAR trafficking. Oxidation of several cytoskeletal‐related proteins closely related to tau was also recovered by MitoQ, such as the beta 3 and 4a tubulins, which are components of the microtubules, together with the cytoplasmic dynein, myosin 10, or the microtubule‐associated protein 1B (MAP1B), which might affect axonal transport. Importantly, MAP1B has been shown to modulate synaptic transmission by regulating AMPAR^61^, ^62^, ^63^ and NMDAR trafficking.^64^ Clathrin heavy chain oxidative modifications were also reduced by MitoQ in the patients’ cells. This protein has a major role in the formation of coated vesicles essential for clathrin‐mediated endocytosis, which is implicated in the AMPA^65^, ^66^, ^67^ and NMDA^68^, ^69^ receptors’ internalization. Another component of this machinery, the heat shock cognate 71, Hsc70, a constitutively expressed heat shock protein important for the uncoating of the clathrin‐coated vesicles,^70^ was also recovered by MitoQ. Alterations in Hsc70 might lead to the impairment of clathrin‐mediated endocytosis,^71^ and therefore, AMPAR and NMDAR internalization. Interestingly, oxidative modifications of this protein were also found in AD brains by redox proteomics.^59^ In addition, the tyrosine‐protein phosphatase Shp‐2 also has a role in the regulation of NMDA functionality, by regulating its phosphorylation.^72^ Taken together, redox proteomics data show that MitoQ was able to reverse the oxidative modifications found in several proteins implicated in AMPAR and NMDAR trafficking in patients’ cells, suggesting this could be the mechanism for the altered glutamatergic signaling induced by mitochondrial ROS.

Here, we show that tau pathology alters glutamatergic signaling, and, conversely, it has also been described that overactivation of AMPARs and NMDARs are able to trigger tau hyperphosphorilation and pathology.^73^, ^74^ Increased neuronal activity also leads to enhanced tau secretion and propagation.^45^ We show that iPSC‐derived neurons are able to secrete tau, and importantly, extracellular tau (specifically the 4R isoform, either patient‐secreted or the recombinant K18 fragment) reproduces the alterations in the glutamatergic signaling observed in the mutated iPSC neurons. Rather than a direct effect of extracellular tau in the receptors at the membrane site, the mechanism appears to be also mediated by tau‐induced mitochondrial ROS, as the altered calcium signaling is restored in the presence of mitochondrially targeted (intracellular) antioxidants. Thus, this could be one of the mechanisms for tau pathology propagation and amplification in FTD and other tauopathies.

In summary, our results show that mitochondrial ROS induced by 4R tau pathology mediate glutamatergic signaling alteration via AMPA and NMDA receptors in iPSC‐derived neurons and primary neuronal cultures. Mitochondrial antioxidants are able to prevent both the altered calcium signaling and the induced neuronal death, offering a new therapeutic strategy for indirect glutamatergic modulation in tauopathies. Importantly, this type of compounds have already been shown to prevent cognitive decline in mice models of dementia.^75^, ^76^

## CONFLICTS OF INTEREST

The authors declare no competing interests.

## AUTHOR CONTRIBUTIONS

Conceptualization: Andrey Y. Abramov, Noemí Esteras. Investigation and analysis: Noemí Esteras, Olga Kopach, Marta Maiolino, Morana Jaganjac. Resources: Seema Qamar, Dmitri A. Rusakov, Morana Jaganjac, Andrey Y. Abramov, Selina Wray. Writing—original draft: Andrey Y. Abramov, Noemí Esteras. Writing—reviewing and editing: Andrey Y. Abramov, Noemí Esteras, Olga Kopach, Morana Jaganjac, Dmitri A. Rusakov. Visualization: Noemí Esteras, Olga Kopach. Funding acquisition: Vincenzo Lariccia, Salvatore Amoroso, Dmitri A. Rusakov, Morana Jaganjac, Andrey Y. Abramov.

## Supporting information

SUPPORTING INFORMATION
