# Supplementary material for: Mitochondrial ROS control neuronal excitability and cell fate in frontotemporal dementia
Source: Alzheimers Dement. 2021 May 31;18(2):318–38. doi: 10.1002/alz.12394 (PMC12086756; doi:10.1002/alz.12394)
Supplement: Supplementary file 1 — SUPPORTING INFORMATION [file ALZ-18-318-s001.pdf]

SUPPLEMENTARY METHODS

Cytosolic ROS production

Cytosolic ROS production was first monitored in single cells using dihydroethidium (DHE, 2 μM, Molecular Probes, ThermoFisher). Upon oxidation of the dye mostly by superoxide, the indicator intercalates within the DNA, staining the nucleus fluorescent red. Fluorescence was detected using an epifluorescence inverted microscope equipped with a ×20 fluorite objective. Rate of increase in DHE intensity was monitored in single cells using excitation light provided by a xenon arc lamp, the beam passing a monochromator at 530 nm (Cairn Research, Kent, UK). Emitted fluorescence light was reflected through a 605-nm longpass filter to a cooled CCD camera (Retiga; QImaging) and digitized to 12-bit resolution. All imaging data were collected and analysed using software from Andor (Belfast, UK). Additional experiments were done using the general ROS indicator CM-H2DCFA with the same experimental conditions and equipment. Rate of increase in green fluorescence was monitored in single cells using an excitation at 488 nm and a FITC emission filter (ET525/36m).

ELISA

Secreted tau levels were quantified in the supernatant of the iPSC-derived cultures with the Human Tau (total) ELISA kit (Invitrogen) following the manufacturer’s instructions. Samples were collected 48 hours after media change. In some experiments, after media collection, cells were washed twice in cold PBS and protein lysates were prepared using RIPA buffer. Total protein amount was then quantified using the Pierce BCA Protein Assay Kit (Thermo Scientific). This was used to correct the amount of secreted tau by the total amount of protein present in the corresponding well.

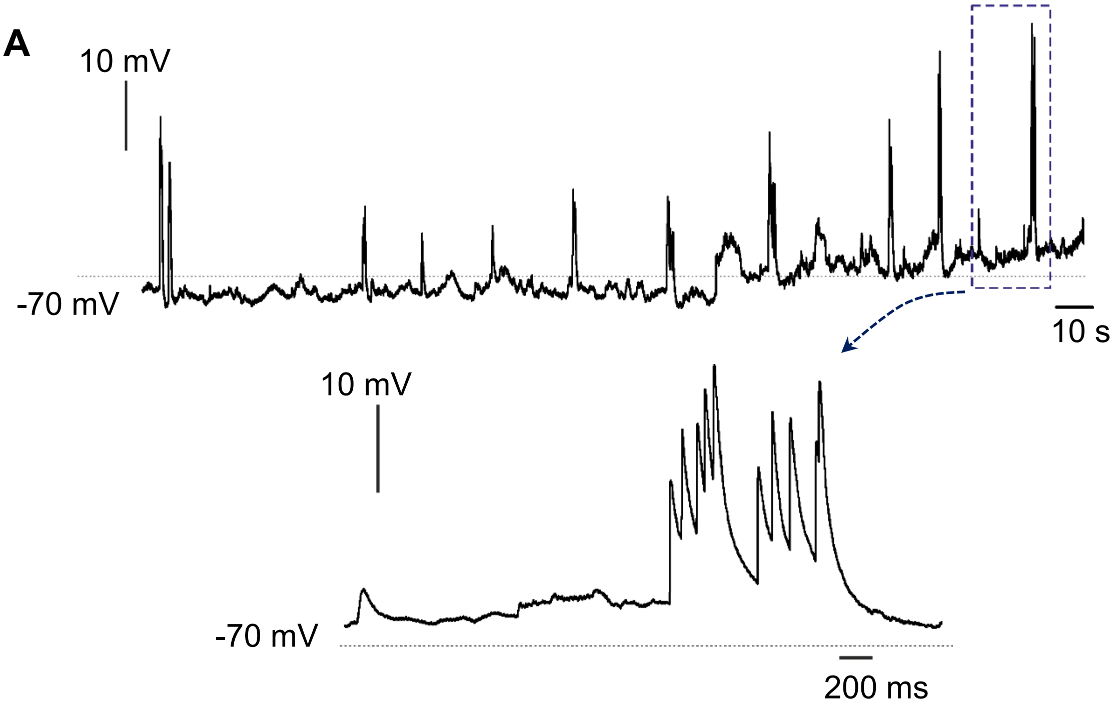

Supplementary Figure 1

Spontaneous activity of human iPSC-derived cortical neurons with the *MAPT* 10+16 mutation.

A. Representative cell-attached recording in an iPSC-derived cortical neuron of the FTD group showing spontaneous changes in the membrane potential. Dotted box shows changes on an expanded scale. Total cells recorded: n = 13.

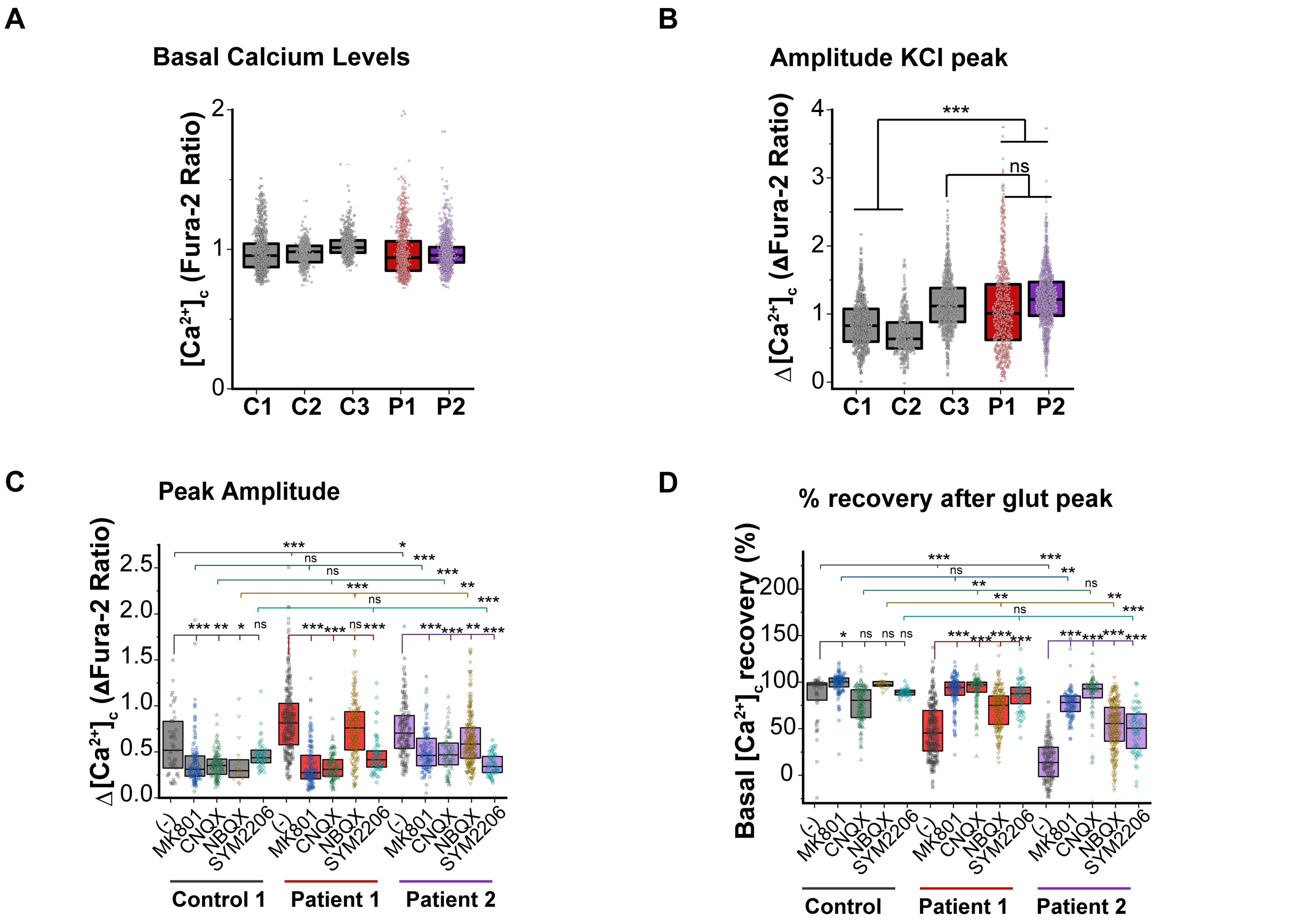

Supplementary Figure 2

Glutamate-induced calcium signalling through AMPAR and NMDARs is increased in iPSC-derived neurons with *MAPT* 10+16 mutation

Cytosolic calcium levels ($[Ca^{2+}]_c$) of iPSC-derived neurons from controls (C1-3) and patients with the 10+16 mutation (P1-2) were analysed by live cell imaging using Fura-2 AM, as depicted in Figure 3.

A-B. *Quantification from the experiments whose representative traces are showed in Fig. 3A* **(A)** Basal cytosolic calcium levels $[Ca^{2+}]_c$ of the neurons before any stimulation as measured by the fura-2 ratio. **(B)** Peak amplitude of the calcium response after the application of 50 mM KCl. C1 n=760, C2 n=470, C3 n=810, P1 n=717, P2 n=1251 neurons. Box plots represent the median, 25 and 75 percentiles; non-parametric Kruskal-Wallis H test, ***p< 0.0001.

C-D. *Quantification from the experiments whose representative traces are showed in Fig. 3F-G.* **(C)** Peak amplitude, and **(D)** percentage of the basal calcium levels recovery after the peak at the end of the glutamate exposure in the presence or absence of the different ionotropic glutamate receptor antagonists. No drug pre-treatment (-) (C, n=49 neurons; P1, n=250; P2, n=152). NMDA antagonist MK801, 10 µM (C, n=125; P1, n=128; P2, n=108). AMPA/kainate antagonist CNQX, 20 µM (C, n=128; P1, n=119; P2, n=80). AMPA-selective competitive antagonist NBQX, 20 µM (C, n=17; P1, n=175; P2, n=233). AMPA-selective non-competitive antagonist SYM2206, 10 µM (C, n=46; P1, n=61; P2, n=61). Box plots represent the median, 25 and 75 percentiles. Non-parametric Kruskal-Wallis H test was used to determine if there were statistically significant differences between controls and patients, or between the treatments in each group (ns: non-significant, *p<0.05, **p<0.01, ***p<0.0001).

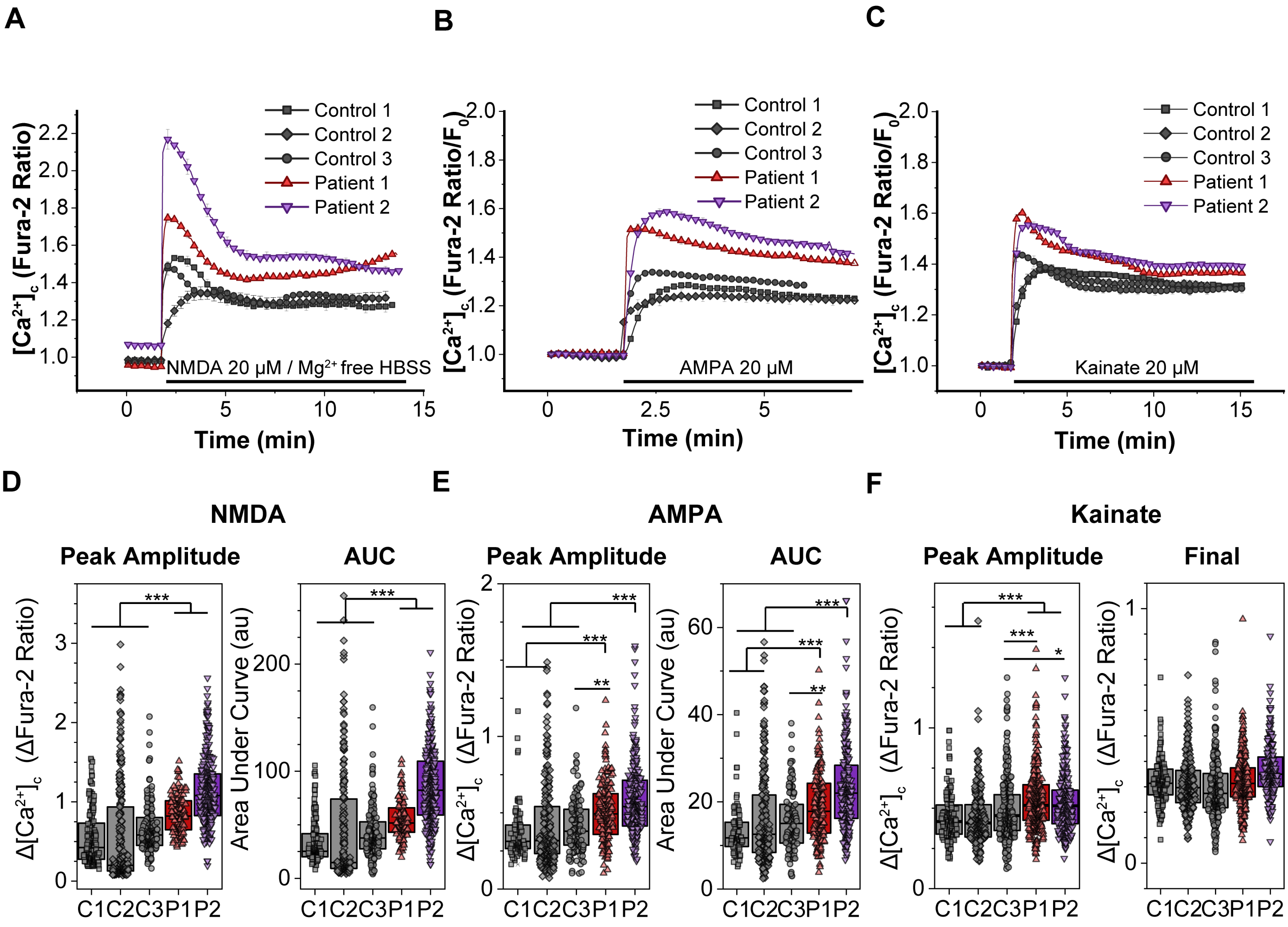

Supplementary Figure 3

Glutamate receptor agonists NMDA and AMPA induce an increased calcium signal in iPSC-derived neurons with *MAPT* 10+16 mutation

A-C. Traces from representative experiments showing the mean ± SE of Fura-2 ratio after the stimulation of controls and patients' neurons with different glutamate receptors agonists: (A) NMDA 20 μM in Mg^{2+}-free HBSS, (B) AMPA 20 μM and (C) kainate 20 μM.

D-E. Peak amplitude and area under the curve for the response induced by the glutamate receptor agonists NMDA 20 μM in Mg^{2+}-free HBSS (D), AMPA 20 μM (E) in controls and patients' neurons. NMDA: C1 n=177, C2 n=268, C3 n=217, P1 n=124, P2 n=292 cells analysed; AMPA: C1 n=96, C2 n=263, C3 n=130, P1 n=173, P2 n=252.

F. Calcium peak and remaining calcium levels at the end of the experiment induced by kainate 20 μM. C1 n=160, C2 n=264, C3 n=280, P1 n=256, P2 n=245 cells analysed.

Box plots represent the median, 25 and 75 percentiles; each dot corresponds to the value of a single neuron. **p*<0.05, ***p*<0.01, ****p*<0.0001, non-parametric Kruskal-Wallis H test

GENETICALLY ENGINEERED *MAPT* IVS10+16 iPSC-DERIVED NEURONS

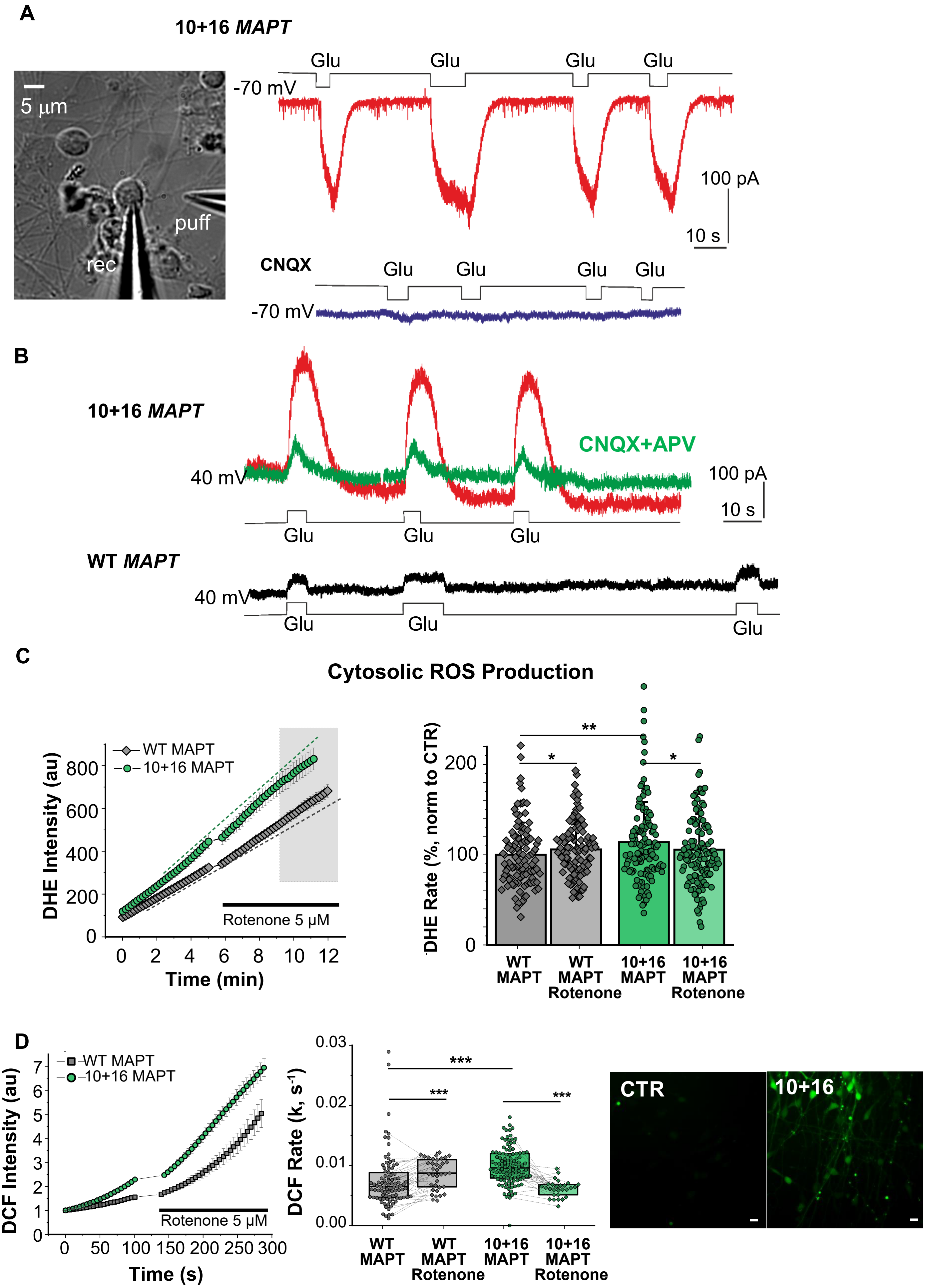

Supplementary Figure 4

Genetically engineered 10+16 *MAPT* iPSC-derived neurons display increased AMPAR/NMDAR-mediated currents and elevated ROS production dependent on hyperpolarized mitochondria.

A-B. Patch-clamp experiments show the increased AMPAR/NMDAR-mediated currents in genetically engineered 10+16 *MAPT* iPSC-derived neurons. **A**. Image, experimental arrangement for a local application of glutamate; recording (rec) and application (puff) pipettes. Traces, AMPAR-mediated currents recorded in a 10+16 *MAPT* iPSC-derived neuron in response to a sequential glutamate puffs (Glu, 100 mM, as shown on the top) at –70 mV, eliminated by CNQX (blue line on the bottom). **B.** Glutamate-evoked currents recorded at 40 mV in a 10+16 *MAPT* iPSC-derived neuron (on the top) and in their isogenic control (WT *MAPT*) neuron (on the bottom) in response to a series of glutamate puffs (Glu, 100 mM, as noted). An AMPA/kainate antagonist CNQX (10 µM, in bath) reduced the current at 40 mV showing the remaining NMDAR-mediated component in a 10+16 *MAPT* neuron.

C, D. Assessment of cytosolic ROS production by DHE and CM-H2DCFDA. **(C)** Cytosolic ROS production was assessed with DHE. Traces from one representative experiment (left) and quantification of the rate of ROS production before and after the application of the inhibitor of complex I rotenone on each neuron. Histograms represent the mean±SD; WT, n=107 neurons; 10+16, n=127. WT *vs.* 10+16, two-sample unpaired t-test, $**p<0.01$. Basal *vs.* rotenone, paired t-test, $*p<0.05$. **(D)** Additionally, cytosolic ROS production was assessed by CM-H2DCFDA imaging. Traces from one representative experiment (left), quantification of the rate of ROS production before and after the application of the inhibitor of complex I rotenone on each neuron (middle) and representative images are shown. WT, n=95; WT+rot, n=37; 10+16, n=112; 10+16 + rot, n=28. $***p<0.0001$, non-parametric Kruskal-Wallis H test. Scale bar: 5 µm.

iPSC-DERIVED NEURONS FROM PATIENTS

A

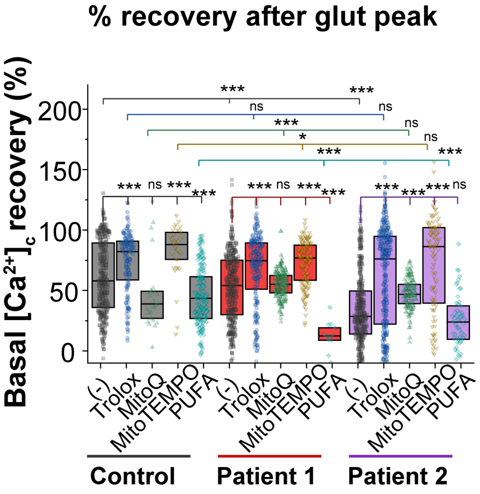

GENETICALLY ENGINEERED *MAPT* IVS10+16 iPSC-DERIVED NEURONS

B

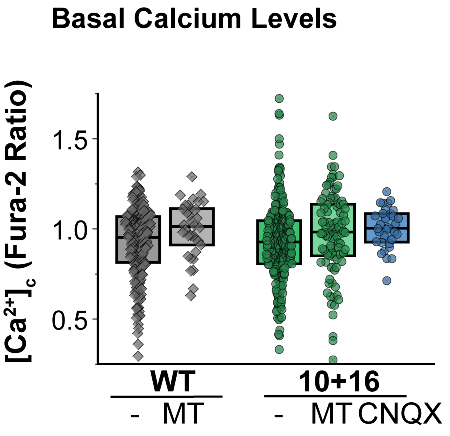

C

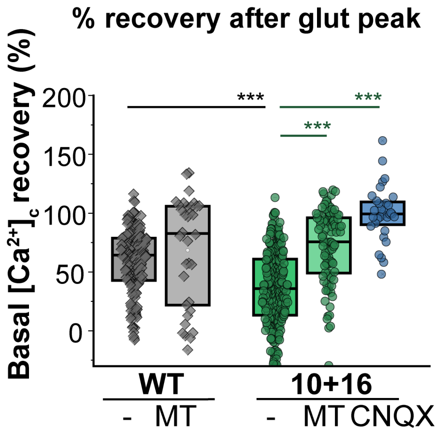

Supplementary Figure 5

Mitochondrially-located antioxidants prevent the alteration of the glutamate-induced calcium-signal in the *MAPT* 10+16 iPSC-neurons

A. *Quantification from the experiments whose representative traces are showed in Fig. 5A* Percentage of the basal calcium levels recovery after the peak at the end of the glutamate exposure (100% represents a full recovery of the calcium basal levels, 0% represents the maintenance of the peak amplitude=no recovery), in the presence or absence of the different antioxidants. No drug (C, n=346 neurons; P1, n=393; P2, n=405); Trolox (C, n=154; P1, n=218; P2, n=336); MitoQ (C, n=26; P1, n=218; P2, n=154); MitoTEMPO (C, n=33; P1, n=140; P2, n=103); d-PUFA (C, n=161; P1, n=13; P2, n=55). Box plots represent the median, 25 and 75 percentiles; Non-parametric Kruskal-Wallis H test was used to determine if there were statistically significant differences between controls and patients, or between the treatments in each group (ns: non-significant, *$p<0.05$, **$p<0.01$, ***$p<0.0001$).

B, C. *Quantification from the experiments whose representative traces are showed in Fig. 5G.* **(B)** Basal cytosolic calcium levels ($[Ca^{2+}]_c$) of the neurons before any stimulation as measured by the fura-2 ratio. **(C)** Percentage of basal calcium levels recovery after the peak at the end of the glutamate exposure (100% represents a full recovery of the calcium basal levels, 0% represents the maintenance of the peak amplitude=no recovery). Box plots represent the median, 25 and 75 percentiles; WT, n=222 neurons; WT+MT, n=34; 10+16, n=279; 10+16+MT, n=97; 10+16+CNQX, n=32. Non-parametric Kruskal-Wallis test, ***$p<0.0001$.

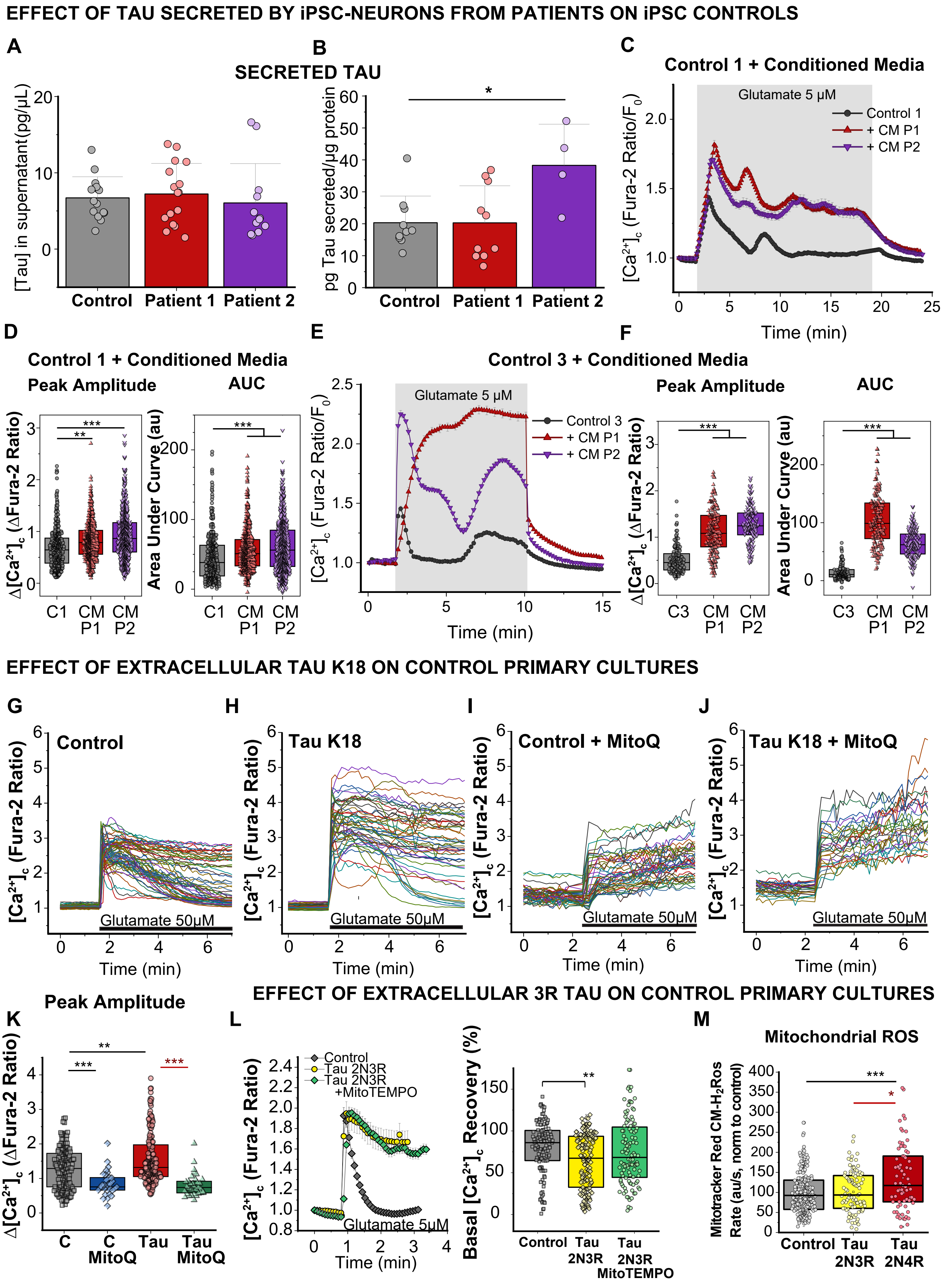
EFFECT OF TAU SECRETED BY iPSC-NEURONS FROM PATIENTS ON iPSC CONTROLS
A
B
SECRETED TAU
[Tau] in supernatant (pg/μL)
Control Patient 1 Patient 2
pg Tau secreted/μg protein
*
C
Control 1 + Conditioned Media
Glutamate 5 μM
Control 1
+ CM P1
+ CM P2
$[Ca^{2+}]_c$ (Fura-2 Ratio/F_0)
Time (min)
D
Control 1 + Conditioned Media
Peak Amplitude
AUC
$\Delta[Ca^{2+}]_c$ (ΔFura-2 Ratio)
Area Under Curve (au)
C1 CM P1 CM P2
E
Control 3 + Conditioned Media
Glutamate 5 μM
Control 3
+ CM P1
+ CM P2
F
Peak Amplitude
AUC
C3 CM P1 CM P2
EFFECT OF EXTRACELLULAR TAU K18 ON CONTROL PRIMARY CULTURES
G
Control
H
Tau K18
I
Control + MitoQ
J
Tau K18 + MitoQ
$[Ca^{2+}]_c$ (Fura-2 Ratio)
Glutamate 50μM
Time (min)
K
Peak Amplitude
$\Delta[Ca^{2+}]_c$ (ΔFura-2 Ratio)
C C MitoQ Tau Tau MitoQ
EFFECT OF EXTRACELLULAR 3R TAU ON CONTROL PRIMARY CULTURES
L
Control
Tau 2N3R
Tau 2N3R +MitoTEMPO
Glutamate 5μM
$[Ca^{2+}]_c$ (Fura-2 Ratio)
Time (min)
Basal $[Ca^{2+}]_c$ Recovery (%)
Control Tau 2N3R Tau 2N3R MitoTEMPO
M
Mitochondrial ROS
Mitotracker Red CM-H_2Ros Rate (au/s, norm to control)
Control Tau 2N3R Tau 2N4R

Supplementary Figure 6

Extracellular tau alters the glutamate-induced response of iPSC and primary control neurons by increasing mitochondrial ROS production

A. Tau concentration in the culture media from control (n=15 independent samples) and mutant iPSC-derived neurons (P1, n=16, P2, n=12) measured by ELISA. **B.** Amount of secreted tau in the extracellular media per µg of total protein in the corresponding cellular extract. Histograms represent the mean ± SD. C, n=10; P1, n=10; P2, n=4, one-way ANOVA, *$p<0.05$.

C-F. Control (1 and 3) iPSC-derived neurons were incubated for 48 hours with the conditioned media (CM) containing secreted tau from each of the patients with the *MAPT* 10+16 mutation (CM P1 or CM P2), and calcium response to glutamate was analyzed. **C, E.** Traces from one representative experiment. **D, F.** Peak amplitude and area under the curve of the calcium response induced by glutamate in the conditions described above. Box plots represent the median, 25 and 75 percentiles; C1, n=423 neurons; C1+CM P1, n=539; C1+CM P2, n=726; C3, n=181; C3+CM P1, n=174; C3+CM P2, n=155. Non-parametric Kruskal-Wallis test, **$p<0.01$, ***$p<0.0001$.

G-J. Representative traces illustrating the glutamate-induced calcium response on rat primary neurons to a pathological (50 µM) concentration of glutamate in control conditions (G), after the incubation of the cells for 24 hours with 300 nM K18 tau (H), and after preincubating the cells for 1 hour with 100 nM MitoQ prior to the experiment (I, J). **K.** Amplitude of the calcium peak in the conditions described before. Box plots represent the median, 25 and 75 percentiles; C, n=190 neurons; C + MitoQ, n=31; Tau, n=200; Tau+MitoQ, n=33. Non-parametric Kruskal-Wallis test, **$p<0.01$, ***$p<0.0001$.

L, M. Cellular effects induced by 3R tau. 2N3R or 2N4R tau as indicated were incubated for 24 h. (L) Representative traces and % of recovery of basal $[Ca^{2+}]_c$ of the calcium responses to 5 µM glutamate are shown. C, n=120 neurons; Tau 2N3R, n=151; Tau+MitoTEMPO, n=108. Non-parametric Kruskal-Wallis test, **$p<0.01$. (M). Rate of mitochondrial ROS production in control, n=230; Tau 2N3R, n=98; Tau 2N4R, n=71 neurons. *$p<0.05$, ***$p<0.0001$. Non-parametric Kruskal-Wallis test.
